# Combined Stochastic and Deterministic Processes Drive Community Assembly of Anaerobic Microbiomes During Granule Flotation

**DOI:** 10.3389/fmicb.2021.666584

**Published:** 2021-05-14

**Authors:** Anna Christine Trego, Paul G. McAteer, Corine Nzeteu, Therese Mahony, Florence Abram, Umer Zeeshan Ijaz, Vincent O’Flaherty

**Affiliations:** ^1^Microbial Ecology Laboratory, Microbiology, School of Natural Sciences and Ryan Institute, National University of Ireland, Galway, Ireland; ^2^Functional Environmental Microbiology, Microbiology, School of Natural Sciences and Ryan Institute, National University of Ireland Galway, Galway, Ireland; ^3^Water Engineering Group, School of Engineering, The University of Glasgow, Glasgow, United Kingdom

**Keywords:** anaerobic digestion, community assembly, low-temperature anaerobic digestion, sludge flotation, dairy wastewater

## Abstract

Advances in null-model approaches have resulted in a deeper understanding of community assembly mechanisms for a variety of complex microbiomes. One under-explored application is assembly of communities from the built-environment, especially during process disturbances. Anaerobic digestion for biological wastewater treatment is often underpinned by retaining millions of active granular biofilm aggregates. Flotation of granules is a major problem, resulting in process failure. Anaerobic aggregates were sampled from three identical bioreactors treating dairy wastewater. Microbiome structure was analysed using qPCR and 16S rRNA gene amplicon sequencing from DNA and cDNA. A comprehensive null-model approach quantified assembly mechanisms of floating and settled communities. Significant differences in diversity were observed between floating and settled granules, in particular, we highlight the changing abundances of *Methanosaeta* and *Lactococcus*. Both stochastic and deterministic processes were important for community assembly. Homogeneous selection was the primary mechanism for all categories, but dispersal processes also contributed. The lottery model was used to identify clade-level competition driving community assembly. Lottery “winners” were identified with different winners between floating and settled groups. Some groups changed their winner status when flotation occurred. *Spirochaetaceae*, for example, was only a winner in settled biomass (cDNA-level) and lost its winner status during flotation. Alternatively, *Arcobacter butzerli* gained winner status during flotation. This analysis provides a deeper understanding of changes that occur during process instabilities and identified groups which may be washed out—an important consideration for process control.

## Introduction

Even Earth’s most extreme environments are teeming with complex microbial communities, performing vital eco-system functions. Microorganisms persist in nearly every environment, both natural and built, regulating global biogeochemical cycling of critical nutrients (Cardinale et al., 2012; Locey and Lennon, 2016). Understanding and measuring biodiversity and the way these complex communities assemble and continue to develop has intrigued ecologists for decades (Gaston, 2000). The use of null-models for identifying and quantifying community assembly has continued to gain traction with several advances in both methodology and interpretation (Presley et al., 2010; Chase et al., 2011; Stegen et al., 2012; Tucker et al., 2016; Zhou and Ning, 2017; Verster and Borenstein, 2018; Ning et al., 2019; Vass et al., 2020). Recently the assembly in rock pool communities was assessed using a combination of null-models, finding that dispersal limitation was a previously underestimated process driving assembly in these communities (Vass et al., 2020). Alternatively, a lottery-based approach was implemented to explore clade-based assembly in the human gut microbiome by identifying lottery “winners” which were out-competing other closely related taxa (Verster and Borenstein, 2018).

Using a combination of several approaches and ecological frameworks, we can begin to piece together the dynamics of assembly processes over space and time, and under varying environmental conditions. These processes include both stochastic and deterministic mechanisms. Stochastic processes include ecological drift driven by random birth-death events and random colonisation. Conversely, deterministic processes are driven by both abiotic (environmental filtering: changes in pH, temperature, salinity, etc.) and biotic factors (competition, facilitation, mutualism, predation, etc.) (Ning et al., 2019). Moreover, we understand that several distinct mechanisms can act on the microbiome simultaneously—especially in complex communities—and that the relative proportion of these processes can change (Stegen et al., 2013; Vass et al., 2020). For example, several studies have identified shifts from stochasticity to determinism following an environmental disturbance (Fritsche and Hofrichter, 2000; Zhang et al., 2016). To date, there are conflicting reports as to the importance of these types of processes in engineered biological systems—complicated by the fact that each study uses a different ecological framework and methodology (Ofiţeru et al., 2010; Zhou et al., 2013; Vanwonterghem et al., 2014; Leventhal et al., 2018; Ali et al., 2019). However, as a whole, the understanding of how communities assemble in ecosystems of the built environment remains under-studied. Yet, they are wide-spread applications of biotechnology, as well as a source of highly replicated microbial communities (Leventhal et al., 2018; Trego et al., 2020).

Anaerobic granules, for example, form through self-immobilisation of bacteria and archaea (Liu and Tay, 2004) and are crucial to the success of high-rate, upflow anaerobic systems such as the upflow anaerobic sludge bed (UASB) bioreactor (Lettinga, 1995). Each individual granule contains the entire microbial community necessary to convert organic pollutants to a methane-based biogas via the anaerobic digestion (AD) process. Granule flotation, however, is a frequently reported problem associated with UASB bioreactors (de Beer et al., 1996; Cuervo-López et al., 1999; Li et al., 2014; Wang et al., 2018) which can lead to washout of active granules and can dramatically reduce system capacity (Yoda and Nishimura, 1997). Furthermore, persistent flotation and washout can lead to severe biomass loss and, eventually, process failure (Chen et al., 2010).

The causes of flotation are several-fold and complex, but a number of studies have reported that the main issue is attachment of biogas bubbles to the biofilm (Alphenaar, 1994; Yoda and Nishimura, 1997; Li et al., 2014). This occurs when biogas within the granule is produced faster than it can be released (Rinzema et al., 1994; de Beer et al., 1996; Hwu et al., 1998; Halalsheh et al., 2005; Campos et al., 2017; Wang et al., 2018; Singh et al., 2019). Interestingly, the archaeal and bacterial composition, and the location of these organisms within the biofilm seems to be an important factor, although again there are conflicting ideas about their role. Some studies report that aggregates with hydrophobic surfaces have a higher affinity for biogas adsorption, thus granules with methanogenic archaea located at the exterior, may be more susceptible to flotation (Daffonchio et al., 1995; de Beer et al., 1996). Conversely, other studies have demonstrated that granules with methanogens located in the interior were more likely to float due to entrapped biogas within the biofilm (Saiki et al., 2002). Finally, others have demonstrated that excessive growth of *Methanosaeta* can lead to filamentous bulking, which entraps biogas and results in flotation (Li et al., 2008). Missing from these microbiome-based explanations is a deeper analysis of the differences between communities of floating and settled granules and the identification and quantification of the exact ecological mechanisms driving community assembly.

Hence, the aim of this study was twofold: (i) to examine several physiological characteristics of both floating and settled aggregates; and (ii) to resolve patterns in the microbial community structure and assembly that may facilitate our understanding of granule flotation. Not only did we uncover significant differences in microbiome diversity and structure between floating and settled aggregates, but we also found that the assembly patterns differed, resulting in a distinct “floating microbiome.”

## Materials and Methods

### Bioreactor Operation and Biomass Sampling

Anaerobic granules were sourced from a full-scale (1,500 m^3^) internal circulation reactor at Carbery Milk Products (CMP; Ballineen, Co., Cork, Ireland). At CMP the granules were used to treat ethanol production wastewater under mesophilic (37°C) conditions. The volatile solids (VS) concentration of the biomass was 91 g VS L^–1^. The granules were 0.8–3.2 mm in diameter, spherical, and black in colour. For this study they were used to inoculate triplicate, laboratory-scale (3.5-L working volume; Supplementary Figure 1) upflow anaerobic bioreactors, which were operated under identical conditions (McAteer et al., 2020). Briefly, the bioreactors were inoculated with 20 g VS L^–1^ of granular biomass. Synthetic dairy wastewater was continuously supplied, consisting of skimmed milk powder (2.5 g COD L^–1^) supplemented with both macro- and micronutrients (Shelton and Tiedje, 1984). The pH was buffered using 1.2 g L^–1^ NaHCO_3_. The operating temperature, controlled using external water jackets, was maintained at 37°C until day 115, when it was decreased for low-temperature operation to 15°C for the following 75 days. This decrease in temperature was to examine the feasibility of low-temperature anaerobic treatment of dairy wastewater, on which we have previously reported (McAteer et al., 2020). Separately, granular biomass was sampled from the settled and floating biomass layers from each of the triplicate bioreactors after 75 days of operation at 15°C, when sludge flotation was frequently observed. Samples for DNA/RNA extractions were immediately flash-frozen in liquid nitrogen and stored at −80°C.

### Physico-Chemical Characterisation

Granule diameter was measured for individual granules using digital callipers (RS Components Ltd, Northants, United Kingdom; accurate to 0.03 mm). The total solids (TS) and VS concentrations of granules (*n* = 3) from both floating and settled biomass were determined using the standard loss-on-ignition technique (APHA, 2005). VS was calculated as a percentage of the biomass wet weight. Settling velocity of granules (*n* = 341) was determined by measuring the time required for a single granule to travel 0.3 m down a clear, acrylic tube filled with deionised water. The settling velocity was the distance divided by the settling time. Density was calculated using Stokes’ law. Long chain fatty acid (LCFA) content from granular biomass collected from the floating and settled layers was determined on a Varian Saturn 2000 GC/MS system (Varian Inc., Walnut Creek, CA) using a method adapted from Neves et al. (2009) with details available in Supplementary Material. Since non-parametric tests are distribution-free tests, to find significant differences between floating and settled granules, we used the Kruskal-Wallis test.

### DNA/RNA Co-extraction and cDNA Synthesis

DNA and RNA were co-extracted from granular biomass collected from floating (*n* = 6 DNA and *n* = 6 RNA) and settled (*n* = 6 DNA and *n* = 6 RNA) layers of the bioreactors (total of *n* = 24 samples). Specifically, two samples were used from each of the three replicated bioreactors to constitute the six for each category. For each sample, nucleic acids from 3 g of wet biomass, consisting of multiple granules, were extracted on ice following the phenol-chloroform based procedure previously described (Griffiths et al., 2000; McAteer et al., 2020). Concentrations were determined using a Qubit fluorometer (Invitrogen, Carlsbad, CA, United States). An aliquot of DNA was stored at −80°C. cDNA was then synthesised from the RNA. DNA was removed using the Turbo DNA-free kit (Ambion—Invitrogen, Carlsbad, CA, United States). PCR, with universal bacterial and archaeal primers 515F and 806R (Caporaso et al., 2011), confirmed the samples to be DNA-free. cDNA was then synthesised using the SuperScript III Reverse Transcriptase Kit (Thermo Fisher Scientific, Waltham, MA, United States). Successful cDNA generation was confirmed by PCR amplification using primers 515F and 806R and cDNA was stored at −80°C.

### Quantitative Real-Time Polymerase Chain Reaction (qPCR)

Bacterial and archaeal domains were separately targeted and quantified using qPCR. Reactions were performed on both DNA and cDNA samples from floating (*n* = 6) and settled biomass (*n* = 6) following the complete description by McAteer et al. (2020) using bacterial primer pair 1369F and 1492R and Taqman probe TM1389F (Suzuki et al., 2000), and archaeal primer pair 787F and 1059R and the Taqman probe TM915F (Yu et al., 2005). Quantitative standard curves used *Escherichia coli* as a representative bacterial isolate and *Methanosarcina barkeri* as the representative archaeal isolate. Final gene copy numbers were determined per gram wet biomass. We used the non-parametric Mann-Whitney test to compare qPCR values between different categories.

### High-Throughput Sequencing

The V4 region of the 16S rRNA gene was amplified using universal bacterial and archaeal primer set 515F and 806R (Caporaso et al., 2011) with indexed barcodes on the forward primer. Nucleic acids were normalised to 20 μg mL^–1^. Normalised samples were combined and run in triplicate on a 2% agarose gel. The ∼300 bp bands were excised and purified using the Wizard SV gel and PCR clean-up kit (Promega, Madison, Wisconsin, United States). Purified PCR products were normalised to 7.1 ng μL^–1^. Sequencing was performed on the Illumina MiSeq platform by the Centre for Genomic Research in the University of Liverpool (Liverpool, United Kingdom). The sequencing data from this study are available through the NCBI database under the project accession number PRJNA616223 with sample information available (Supplementary Table 1).

### Bioinformatics and Statistical Analysis

Abundance tables were generated by constructing amplicon sequencing variants (ASVs) using the Qiime2 pipeline and the DADA2 algorithm (Bolyen et al., 2019) with details given at^1^. A total of 2 175 ASVs from *n* = 24 samples were identified, with summary statistics for reads per sample as follows: (1st Quantile: 30 203; Median: 35 630; Mean: 36 183; 3rd Quantile: 40 885; Maximum: 49 488). Within the workflow, qiime feature-classifier was used to classify the ASVs against SILVA SSU Ref NR database release v.132, and then qiime phylogeny align-to-tree-mafft-fasttree generated the rooted phylogenetic tree. The biom file for the ASVs was generated by combining the abundance table with taxonomy information using biom utility available in qiime2 workflow. In addition, we have removed contaminants such as chloroplasts and mitochondria as is recommended in taxonomy-based filtering of artifacts in the Qiime2 workflow^2^.

Next Picrust2 (Douglas et al., 2019), and its qiime2 plugin^3^ using the parameters –p-hsp-method pic –p-max-nsti 2 in qiime picrust2 full-pipeline, was used to find KEGG enzymes and MetaCyc pathway predictions. Although prediction process is highly dependent on the number of pathways available for the reference genomes, with Picrust2, it is now possible to generate accurate and putative metabolic maps at community level by virtue of its comprehensive database (∼20,000 genomes), which was not possible for its predecessor, Picrust1 (only 2 011 genomes). The algorithm consistently predicts pathways that have greater than 0.8 correlation with the actual pathways observed using shotgun metagenomic equivalents as highlighted by the authors (Douglas et al., 2019). Indeed, only a few (∼30 ASVs) from this study were not observed in the Picrust2 reference database. This increases our confidence on the prediction by virtue of high coverage.

For ensuing statistical analysis, the reads were rarefied to the sample with the minimum number of sample reads (23 510). This yielded a 24 (sample) × 1 829 (ASV) abundance table. Statistical analyses were performed in R (v.3.4.4) using the combined data generated from the bioinformatics as well as meta-data associated with the study (details available in Supplementary Material).

### Null-Modelling

A multi-phasic null-model approach was used for comprehensive, quantitative, insights into the underlying ecological mechanisms driving community assembly. To apply null modelling techniques, we have used the full ASV table as obtained after preprocessing including removal of contaminants such as mitochondria and chloroplasts as is typical in amplicon workflows. Next, we wanted to understand the influence of the environment on microbial community assemblage. For this purpose, we used Nearest Taxon Index (NTI) to explore phylogenetic dispersion in the data. NTI is preferred because of presence of significant phylogenetic signal across short phylogenetic distances (Wang et al., 2013). Additionally, NTI is useful when phylogenetic signal cannot be measures, as was the case in this study due to lack of substantial trait data. Therefore, NTI helped determine whether the community was structured due to strong environmental pressure (local clustering in the phylogenetic tree). The NTI was calculated using mntd() and ses.mntd() functions from the picante package (Kembel et al., 2010). NTI represents the negative of the output from ses.mntd(). Additionally, it quantifies the number of standard deviations that separate the observed values from the mean of the null distribution (999 randomisation using null.model-“richness” in the ses.mntd() function and only considers taxa as either present or absent regardless of their relative abundance). Positive values indicate that species co-occur with more closely related species more frequently than expected by chance, with negative values suggesting otherwise. NTI measures tip-level divergences (putting more emphasis on terminal clades and is akin to “local” clustering) in phylogeny. For NTI, values > + 2 indicate strong environmental pressure, and values <−2 indicate strong competition among species as the driver of community structure.

Next, a stochasticity ratio calculation was implemented for which Jaccard (incidence-based) and Ružička (abundance-based) metrics were applied to determine the normalised stochasticity ratio (NST) based on author recommendations (Ning et al., 2019). Taxa-Richness constraints of proportional-proportional (P-P) and proportional-fixed (P-F) were applied for each metric. To obtain significance for NST between treatments, we have used permutational multivariate ANOVA (henceforth referred to as PANOVA), as recommended by the authors (Ning et al., 2019).

Next, the quantitative process estimates (QPE) method was used. This is based on an ecological framework that describes assembly processes in terms of selection (variable or homogenous), dispersal (dispersal limitation, or homogenising dispersal) or “undominated” mechanisms (Vellend, 2010; Stegen et al., 2015). Variable selection gives rise to high compositional differences in community structure due to different selective environmental conditions, while homogenous selection is when unchanging environmental conditions result in consistent selective pressure. Dispersal processes refer to the movement of organisms throughout space. High rates of dispersal result in similar communities, referred to as homogenising dispersal. Conversely, dispersal limitation increases differences in community composition resulting in more dissimilar communities. Conceptually, it occurs when low dispersal rates result in a high community turnover (Stegen et al., 2015). It is dispersal limitation which drives ecological drift (stochastic assembly).

The advantage of QPE is that it considers abundances as well as phylogeny of the ASVs. Following the previously described method (Stegen et al., 2015; Bottos et al., 2018), deviation from the observed βMNTD (β-mean-nearest-taxon-distance) and the mean of the null distribution was evaluated using βNTI (β-nearest-taxon-index). When the observed value of βMNTD deviated significantly from the null expectation, the community was assembled by variable (βNTI >+2) or homogenous (βNTI <−2) selection processes. If the difference was not significant, the observed differences in phylogenetic composition were considered to be the result of dispersal mechanisms enabling ecological drift. These were differentiated using the abundance-based β_RC_ and a Bray-Curtis dissimilarity metric for beta diversity. If the β_RCbray_ > + 0.95, assembly was by dispersal limitation coupled with drift; if β_RCbray_ < −0.95 then homogenising dispersal mechanisms contributed to community assembly; and if β_RCbray_ was between −0.95 and + 0.95, community turnover was due to undominated mechanisms (i.e., dominated neither by dispersal nor selection processes).

Finally, we applied the competitive lottery model for clade-based community assembly (Verster and Borenstein, 2018). Briefly, the model describes the abundance of the most prevalent ASVs in the samples according to a group-based competitive lottery schema. Groups/clades are categorised phylogenetically, in our case at the family-level. It assumes that phylogenetically similar ASVs will have a similar gene content, metabolism, and preferential niche space—all resulting in high levels of competition. Lottery “winners” were defined as a member (ASV) that captures >90% of the groups abundance, and were identified. Families which included winners were plotted based on winner prevalence (the fraction of samples which include a winner ASV for that family) and winner diversity (the frequency that each ASV occurs as the winner in the samples from which winners are observed). A low diversity suggested that the same ASV was dominating that family in all samples while a high diversity suggested a more even spread of ASVs as winners in that group.

## Results

In this study we sampled floating and settled granules from three replicated, low-temperature, laboratory-scale UASB bioreactors from one timepoint, when all three reactors were experiencing the flotation phenomenon. Both floating and settled biomass was compared in terms of density, settling velocity, LCFA composition and aggregate size. Additionally, the makeup of the microbial community was analysed by both qPCR and sequencing the 16S rRNA genes from DNA and cDNA of both floating and settled granules. A comprehensive range of multivariate analysis and null-models provided a novel way of understanding the differences between floating and settled microbial communities.

### Characterisation of Floating and Settled Granules

**FIGURE 1:**
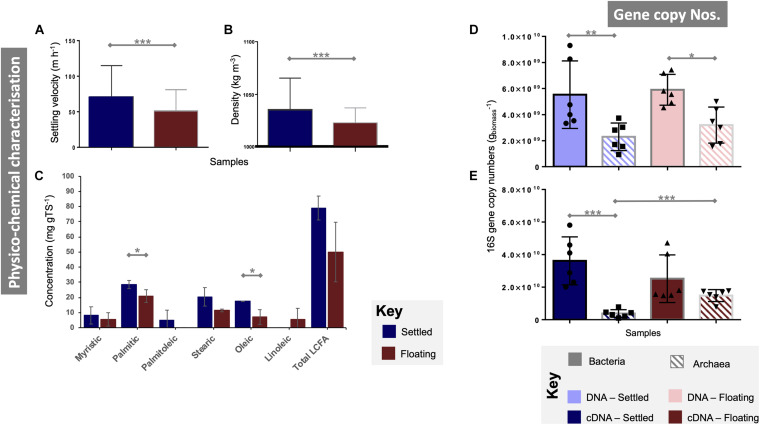
Characterisation of floating and settled granules according to **(A)** the settling velocity (*n* = 341); **(B)** density (*n* = 341); **(C)** concentrations of a range of long-chain fatty acids (LCFA; *n* = 3); bacterial and archaeal qPCR gene copy numbers from **(D)** DNA (*n* = 6) and **(E)** cDNA (*n* = 6). Lines for (**A–E)** connect two categories where the differences were significant with *(*p* < 0.05), **(*p* < 0.01), or ***(*p* < 0.001).

Overall, floating and settled fractions had similar size distributions. Settled granules ranged in diameter from 0.2 (the smallest granules sampled) to 4.12 mm. Floating granules were only slightly smaller ranging from 0.2 to 3.89 mm. The mean granule diameter for floating and settled biomass was statistically similar (*p* = 0.73; Supplementary Figure 2). Settled granules displayed significantly higher settling velocities (*p* < 0.0001) and densities (*p* < 0.0001; Figures 1A,B). Total LCFA analysis showed large differences between floating and settled biomass, but a high standard deviation for the floating granules (±19.7) demonstrated high variability in LCFA concentrations from that category (Figure 1C), resulting in no significant differences (*p* = 0.13). Statistically significant differences were observed, however, for two of the individual LCFA (stearic acid, *p* = 0.05; oleic acid, *p* = 0.05), with higher concentrations in the settled biomass.

DNA-based gene copy numbers revealed significantly higher numbers of bacteria than archaea in both settled (*p* < 0.005) and floating (*p* < 0.05) biomass (Figure 1D). However, they did not differ statistically *between* floating and settled biomass. Gene copy numbers from cDNA showed similar gene copy numbers for bacteria in floating and settled granules, but detected significantly (*p* = 0.0001) higher numbers of archaea in the floating granules. Indeed, archaeal means approached 1.5 × 10^10^ and 3.8 × 10^9^ for floating and settled biomass, respectively (Figure 1E).

### Microbiome of Floating and Settled Granules

**FIGURE 2:**
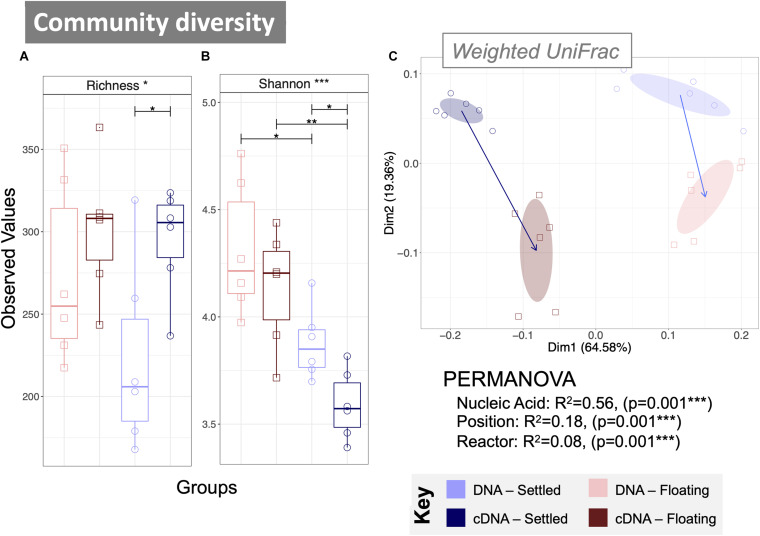
Microbial community diversity according to variances in 16S rRNA genes from both DNA and cDNA (*n* = *6*) measured using alpha diversity measures: **(A)** rarefied richness and **(B)** Shannon Entropy; and beta diversity using principle coordinate analysis (PCoA) with **(C)** weighted UniFrac distances. Samples are indicated by colour, and the ellipses are drawn at a 95% CI for all samples from each category, where arrows mark the direction of change in the community structure from mean ordination of settled samples to the mean ordination of floating samples for each nucleic acid type—the length indicating the amount of change. PERMANOVA (distances between groups) indicate significant differences according to nucleic acid, position and reactor. Lines for **(A,B)** connect two categories where the differences were significant (ANOVA) with *(*p* < 0.05), **(*p* < 0.01), or ***(*p* < 0.001).

The rarefied richness (numbers of ASVs) was statistically similar between floating and settled granules (Figure 2A), while the balance of the community, measured as Shannon Entropy, was significantly lower in settled granules. This was observed in both DNA (*p* < 0.05) and cDNA (*p* < 0.01) samples (Figure 2B and Supplementary Figure 3). This suggests that while the distribution of the community was different, the actual number of observed taxa remained stable.

Significant differences in community composition were observed between floating and settled samples for both the total (DNA-based) and active (cDNA-based) communities using weighted UniFrac distances (Figure 2C; see S4 for unweighted UniFrac). Indeed, PERMANOVA confirmed that the nucleic acid and position of the biomass (i.e., whether it was floating or settled) significantly contribute to (*p* = 0.001) the variance between categories. Additional differences in diversity were observed between the total and active communities, which clustered separately (Figure 2C).

**FIGURE 3:**
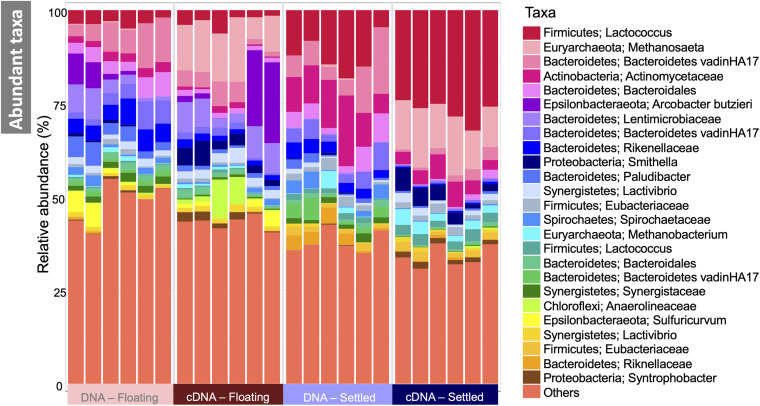
Microbial community in floating and settled granules according to variance in the 16S rRNA genes depicted as a stacked bar chart of the relative abundance of the 25 most abundant taxa (named first by Phylum and second by the most refined taxonomic classification available), and where “others” represent everything that is not in the top-25.

**FIGURE 4:**
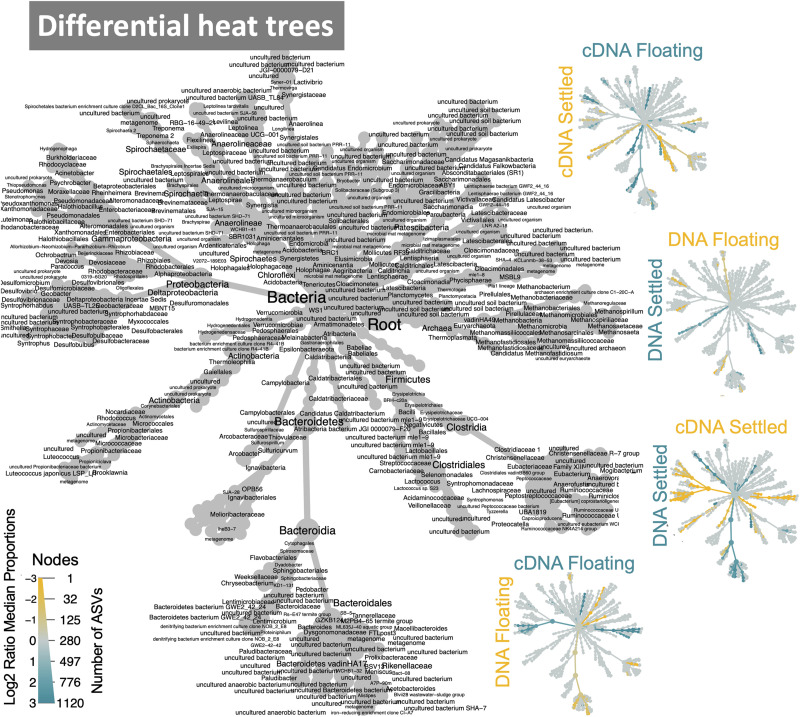
Dynamic taxa between floating and settled granules according to variances in the 16S rRNA genes where differential heat trees highlight clades that are enriched between two groups and label colour indicates the category where the clade is more abundant; reference tree in grey.

The makeup of the most abundant (top-25) taxa in the microbiome across all samples showed several interesting differences between floating and settled biomass (Figure 3). Notably, *Lactococcus* was relatively more abundant in settled biomass as opposed to floating. However, the relative abundance of fermentative bacteria, in general, fluctuated between the two categories. *Anaerolineaceae*, *Lentimicrobiaceae*, and *Arcobacter* were more relatively abundant in the floating granules compared to settled. Heat-trees identified entire clades within the microbiome which were enriched between categories (Figure 4). Comparison of the active communities (cDNA) of floating and settled biomass revealed that while the settled biomass had enriched groups of *Methanobacterium*, *Clostridia*, *Actinobacteria*, and *Alphaproteobacteria*, the floating biomass contained active clades of *Methanomicrobiales*, *Bacteroidetes*, *Campylobacteria*, and *Gammaproteobacteria*. Additionally, differences between DNA and cDNA samples were observed. Notably, the *Euryarchaeota* and *Proteobacteria* were enriched in the cDNA more than the DNA suggesting that they have a strong, potentially active role in the community. However, while many groups were dynamic, a core microbiome (Supplementary Figure 5) containing multiple taxa across all of the critical trophic groups required for complete AD persisted (spanning hydrolysis, acidogenesis, acetogenesis, and methanogenesis). This highlights the functional redundancy of the AD microbiome.

**FIGURE 5:**
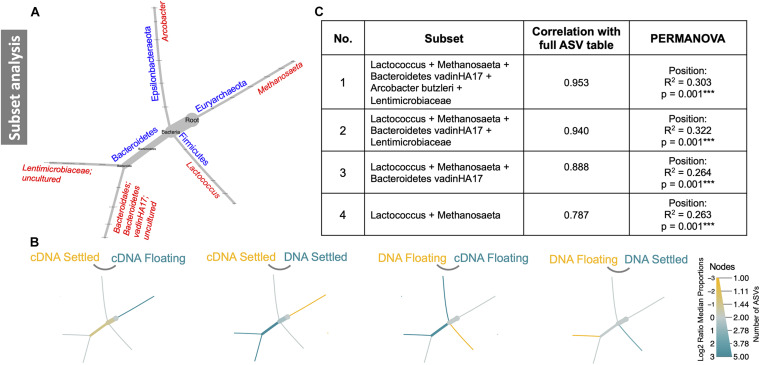
Subset analysis of a subset of five ASVs. These five ASVs are depicted in reduced phylogenetic heat trees where **(A)** is the reference tree; **(B)** highlights branches that were enriched between the two given categories, where colour indicates the category of high abundance; and **(C)** shows four combinations of the five subset ASVs and how they contribute significantly to differences in the microbiome.

Subset analysis was used to identify a minimal group of ASVs, which in several combinations, could statistically explain the observed differences in community structure. Remarkably, only five ASVs (*Lactococcus*, *Methanosaeta*, *Bacteroidetes vadinHA17*, *Arcobacter butzleri*, and *Lentimicrobiaceae*), grouped in four different combinations (Figure 5A), explained between 26 and 30% of variation between floating and settled granules. A focus on the effects these ASVs have on the community structure and how they differed between settled and floating biomass could help explain flotation. For example, heat tree analysis of only these five ASVs revealed that when comparing cDNA of floating and settled biomass, *Methanosaeta*, a methane-producing archaeon, was enriched in the floating biomass. Additionally, *Lactococcus*, which was previously identified as a bacterium of interest (based on relative abundance; Figure 3), was further linked to the settled biomass (Figure 5).

Regressions of environmental/physico-chemical parameters against various diversity measures (Supplementary Figure 6) revealed that biomass position consistently had a significant, and complimentary influence on community structure—floating granules were consistently positively correlated with increased diversity. Notably, archaeal gene copy numbers were also positively correlated with diversity.

Putative functionality, based on Picrust2 algorithms was assessed. Differential pathway analysis was used to identify pathways and kegg orthologues which were significantly enriched between floating and settled biomass (Supplementary Figures 7–9). DNA and cDNA from each position identified 14 such pathways for each nucleic acid type (Supplementary Figures 7, 8). Putative differential pathways based on the DNA, were nearly exclusively (bar one) upregulated in the floating biomass (Supplementary Figure 8). Notably, the mevalonate pathway III, exclusive to the archaea, was putatively enriched in floating biomass (cDNA-based analysis), while pathways pertaining to lactose, or lactate were enriched in the settled biomass.

### Community Assembly Processes

**FIGURE 6:**
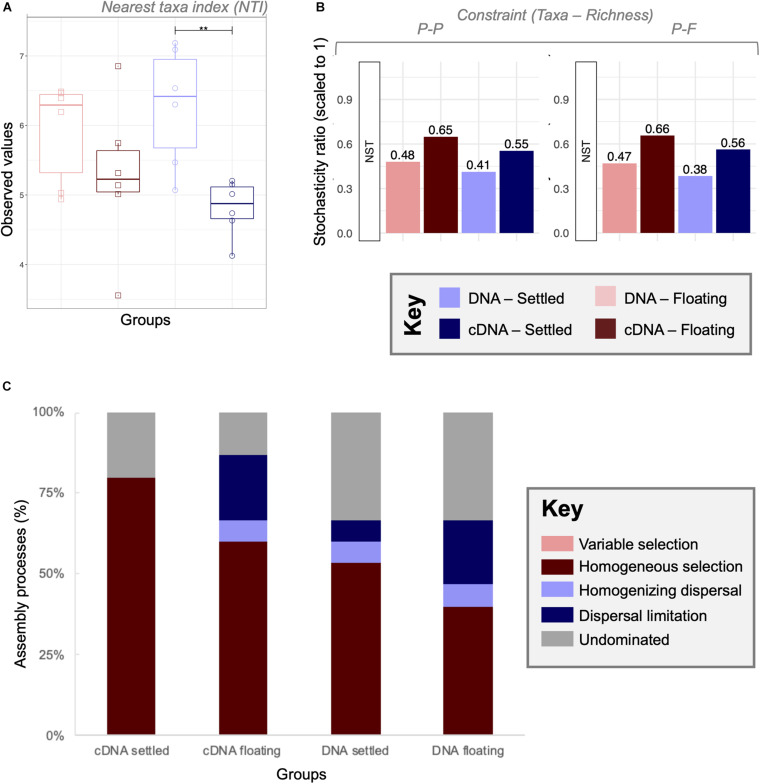
A combined null-model approach to identify and quantify ecological community assembly processes between floating and settled granules using **(A)** environmental filtering calculated as nearest taxa index (NTI) where values > + 2 indicate extreme clustering in the phylogenetic tree driven by environmental pressures (determinism); **(B)** the stochasticity ratio approach, which quantified stochasticity using the Jaccard metric and Taxa-Richness constraints of proportional-proportional (P-P) and proportional-fixed (P-F) as a normalised stochasticity ratio (NST); and **(C)** the quantitative process estimates (QPE) approach which determines the proportion of assembly mechanisms acting on a category within the framework of selection, dispersal and undominated, represented by a stacked bar chart. Lines for **(A)** connect two categories where the differences were significant (ANOVA) with **(*p* < 0.01).

Through NTI analysis, strong environmental pressure was observed for all four categories although no significant changes between floating and settled biomass was detected (Figure 6A). In general, the observed values were lower in cDNA than in DNA, with significant (*p* < 0.01) differences in nucleic acid type for the settled biomass.

Stochasticity was quantified using the normalised stochasticity ratio (Figure 6B). In this case floating and settled categories showed differences in the percent stochasticity. According to NST (P-F), stochasticity accounted for 47–66% of assembly for floating granules, and 38–56% for settled communities. However, none of the differences between floating and settled biomass were significant (*p* = 0.09; PANOVA). The combination of both of these approaches showed that stochastic processes were relevant, but not the only ecological mechanism shaping the microbiome.

To further quantify and identify assembly processes, we used the QPE approach (Figure 6C) which is based on an ecological framework defined by *selection* (deterministic processes), *dispersal* (stochastic processes resulting in ecological drift), and *undominated* mechanisms. Here we observed that undominated processes (neither selection driven, nor dispersal driven) accounted for 13–33% of community turnover. Variable selection played no role, which is explained by the identical environmental conditions of the samples (same temperature, pH, salinity, etc.). Homogeneous selection was the dominant assembly mechanism in all categories, ranging from 40 to 80%. Clear differences in assembly processes were observed between settled and floating categories. Dispersal limitation from cDNA samples increased in relative importance from 0 to 20% between settled and floating biomass, respectively.

**FIGURE 7:**
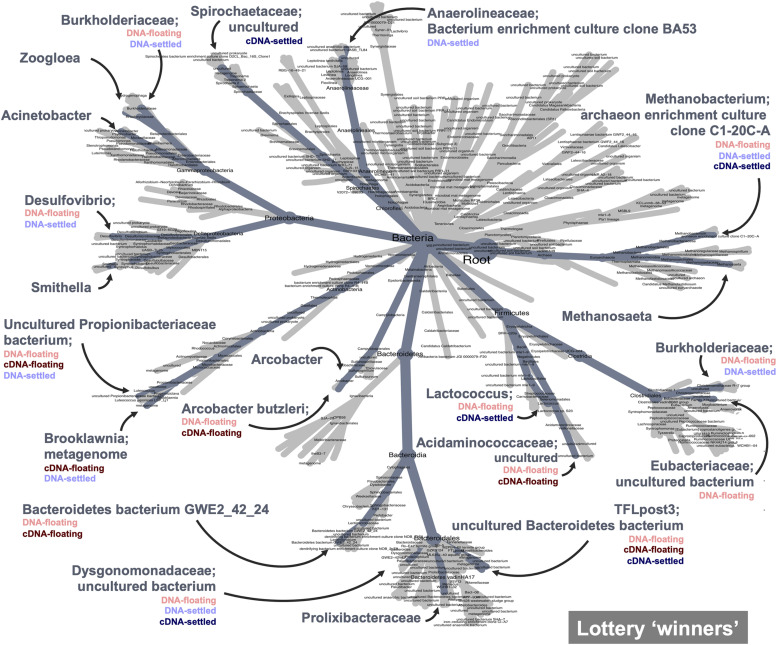
Competitive lottery model for clade-based assembly which identified “winning” ASVs (ASVs with >90% abundance within their defined clade) from family-level clades. Winners are highlighted on the heat tree; categories where winners were identified are listed below winner name; winners with unlisted groups represent winners that won across all categories and therefore flotation had no effect on winner behaviour.

Finally, the lottery model used a different lens to assess clade-based community assembly. Lottery “winners” were identified (those making up >90% of community abundance within their clade) and were plotted based on winner prevalence and diversity (Supplementary Figure 11; genus-level analysis in Supplementary Figure 12). Notably, most families showing lottery-behaviour had low diversities, indicating that only one ASV was dominating as the winner. Two families showed intense lottery-like behaviour—having both high prevalence and diversity, indicating that winners were found in nearly all samples and that several different ASVs were selected as the winners. Most interesting, however, is the difference in winner behaviour between floating and settled samples (Figure 7). The lottery tree highlights winning clades to their most refined taxonomic level, also identifying clades with no lottery behaviour. Clades with listed groupings highlight those where lottery behaviour is changing between groups (i.e., where not all groups were identified for that clade). For example, *Smithella* was a winner in all groups, and thus lottery assembly did not differ due to flotation (no groups listed). *Spirochaetaceae*, however, was only a winner in settled biomass (cDNA) and lost its winner status during flotation. Conversely, *Arcobacter butzerli* gained winner status in the “floating microbiome.”

## Discussion

### Ecological Mechanisms Underpinning Granule Flotation

The combination of null models used in this study revealed that both stochastic and deterministic processes simultaneously drive community assembly—an idea that could reconcile conflicting reports from built environments (Ofiţeru et al., 2010; Zhou et al., 2013; Vanwonterghem et al., 2014; Leventhal et al., 2018; Ali et al., 2019). QPE analysis took this one step further to classify and quantify these processes in terms of selection and dispersal. Interestingly, homogenous selection was the most dominant ecological principle for all categories—where selection refers to ecological fitness and ongoing abiotic and biotic interactions shaping the community. The lottery model additionally suggested that these selection processes may have been governed by intense competition between species.

According to QPE, dispersal processes have a stronger role to play in floating granules. In view of homogeneous selection being a dominant process, as well as dispersal limitation, which was observed in floating samples, we propose the following hypotheses: (i) a lack of connectivity between positions (floating and settled) may result in high compositional turnover due to dispersal limitation and drift; or, (ii) that environmental selection pressure (non-optimal temperatures, pH fluctuations, mixing strategies, etc.) may make it harder for fermenters such as *Lactococcus* to colonise, even when there is connectivity and that these uncolonised granules may eventually float. Moreover, these findings add to the growing series of literature which identifies dispersal limitation as an important factor driving community turnover across various ecosystems (Stegen et al., 2013, 2015; Bottos et al., 2018; Vass et al., 2020). It should be noted that typically a lack of phylogenetic signal leads to non-significant βNTI (required for QPE analysis). The observed significant values of βNTI supports the *post hoc* assumption of phylogenetic signal across short distances, which in turn, increases confidence in the results.

Finally, the lottery model for clade-based assembly identified lottery “winners” across a wide range of the microbiome. Interestingly, clades displaying winner behaviour generally showed low winner diversity indicating that within those clades only one ASV was dominating as the winner. Such organisms are deemed to be out-contending other members of their clade, fiercely competing for available nutrients within their niche space. The strongest lottery-like groups, however, have a high diversity and high winner prevalence (Verster and Borenstein, 2018), indicating a more variable selection of ASVs as winners within the clade. In the context of flotation, changing lottery behaviour gives key insights into how the “floating microbiome” is shaped. Several winners were present in both floating and settled aggregates. Such winners can be thought of as strong competitors regardless of flotation, and are perhaps more resilient to such eco-system disturbances. Winners, however, which are only present in either floating or settled granules indicated changing clade-based assembly mechanisms. *Spirochaetaceae* was a lottery winner (cDNA) in settled granules, but lost its winner status in the floating biomass. This suggests that the winners within *Spirochaetaceae* were not as fit in the new floating biomass. Conversely, some members of other families, such as *Acidaminococcaceae*, found their niche, out-competing the rest of the clade in the floating biomass. Such groups, in particular, give rise to the distinct “floating microbiome.”

### A Distinct “Floating Microbiome”

The microbial communities of floating and settled granules were significantly different from one another. It is possible that the microbial community may play a contributing role in causing the observed reduction in density. Such ideas have been previously explored, for example, the role of methanogenic archaea (Saiki et al., 2002), specifically regarding the acetoclastic genus *Methanosaeta*. Here, *Methanosaeta* was identified during subset analysis, as one of only five ASVs, which in several combinations, statistically explain variances in community structure. Notably, cDNA analysis identified the enrichment of *Methanosaeta* in floating granules (Figure 5), compared to settled. Furthermore, exclusively archaeal pathways, such as the mevalonate pathway—responsible for the synthesis of isoprenoid compounds which are found in archaeal lipid membranes, fundamentally distinguishing them from bacteria—were putatively enriched in the floating granules. This pathway is presumed to be widespread amongst archaea, but has been identified amongst several methanogens (Kazieva et al., 2017; Yoshida et al., 2020). Finally, archaeal gene copy numbers were significantly higher in floating granules according to cDNA analysis. The combination of these analyses suggests that *Methanosaeta* may be linked to flotation.

There are two likely mechanisms by which *Methanosaeta*, and other gas-producing organisms, may contribute to flotation. Both relate to the position such organisms occupy within the biofilm (i.e., on the surface, or in the interior). The primary hypothesis relates to the density; it is possible that granules having relatively higher numbers and/or proportions of active biogas producers in the biofilm interior were generating more biogas. These granules were therefore more likely to have trapped gas pockets, decreasing the biofilm density and leading to flotation. An alternative hypothesis suggests that although methanogenic archaea (primary gas-producing organisms in AD processes) are generally accepted to be located in the core of the granule (MacLeod et al., 1990; Sekiguchi et al., 1999) they can also occupy the surface layers (Saiki et al., 2002). Previous work has explored how a hydrophilic coating of extracellular polymeric substances (EPS) can protect granules from flotation (de Beer et al., 1996). However, in our case, if hydrophobic *Methanosaeta* (Daffonchio et al., 1995; de Beer et al., 1996) occupied biofilm surface, they may contribute to flotation.

### Flotation Linked to Density and Not LCFA

Flotation is a serious problem for AD, resulting in the loss of active biomass, reducing system capacity (Yoda and Nishimura, 1997; McAteer et al., 2020) and in severe cases, resulting in process failure (Chen et al., 2010). The phenomenon is complex—and likely to be influenced by several factors. Flotation has been observed in many cases and many bioreactor types, with several reported causes including reduced biofilm density (Lettinga et al., 1983; Batstone and Keller, 2001; Lu et al., 2012, 2015) sometimes attributed to trapped biogas (Alphenaar, 1994; Yoda and Nishimura, 1997; Chen et al., 2010; Li et al., 2014; Campos et al., 2017), the influence of the microbiome (Daffonchio et al., 1995; de Beer et al., 1996; Saiki et al., 2002; Li et al., 2008) or the influence of LCFA (Rinzema et al., 1994; Hwu et al., 1998; Halalsheh et al., 2005; Singh et al., 2019). Specifically, several studies have attributed flotation to LCFA accumulation, especially when treating lipid-rich wastewaters such as dairy wastewater (Rinzema et al., 1994; Hwu et al., 1998; Vidal et al., 2000; Alves et al., 2009; Singh et al., 2019; Eftaxias et al., 2020). It is generally accepted that due to inherent hydrophobicity, the sorption of LCFA to the granule exterior contributes to flotation (Hwu et al., 1998). The results from our study indicate, however, that LCFA were unlikely to be the primary cause of flotation as more LCFA were found in the settled biomass than in the floating biomass. The findings from the physico-chemical characterisation revealed only that density and settling velocity were significantly reduced in the floating biomass, as other studies have previously reported (Lettinga et al., 1983; Yoda and Nishimura, 1997; Batstone and Keller, 2001; Lu et al., 2012, 2015). This could likewise be due to trapped biogas in the interior of the biofilm.

### Implications for Low-Temperature System Management

Flotation likely has several root causes, but always results in process instabilities including, but not limited to, biomass washout. The microbial community structure of floating and settled granules from this study differed significantly, as did the putative functional diversity and capacity at each position. This suggests that if floating biomass is preferentially washed out of the system, several groups, with particular functions may over time, decline in abundance. Namely, *Anaerolineaceae*, *Arcobacter*, *Lentimicrobiaceae*, along with high numbers of methanogens, were all identified in the floating biomass. Anaerobic microbiomes are known to be functionally redundant (Campanaro et al., 2019; Zhu et al., 2020) however, the sustained loss of multiple key, and potentially active groups may still negatively impact system performance, especially for sensitive, low-temperature operation (McAteer et al., 2020). For example, *Anaerolineaceae* are a family of metabolically diverse bacteria, capable of semi-syntrophic primary, and secondary fermentations (Sekiguchi et al., 2003; Narihiro et al., 2012, 2015) and may further be important for granular structure (Yamada et al., 2005). They have been previously identified as key players during low-temperature AD (Keating et al., 2018) and their reduction could disrupt low-temperature AD eco-system function. Finally, for systems optimising carbon transformations culminating in the production of methane-based biogas, decreased numbers of methanogenic archaea could have severe implications and a devastating impact on system performance (Demirel and Scherer, 2008). Whilst this study was performed on laboratory-scale bioreactors, microbial ecology of full-scale systems is slightly more complex with respect to substrate composition, strength and loading rate, and feeding and heating regimes, as per our previous study (Connelly et al., 2017). Therefore, it is plausible to expect full-scale ecology to be slightly more dominated by selection processes than under controlled laboratory conditions.

## Data Availability Statement

The datasets presented in this study can be found in online repositories. The names of the repository/repositories and accession number(s) can be found below: https://www.ncbi.nlm.nih.gov/, PRJNA616223.

## Author Contributions

PM, VO’F, and FA designed the study. PM performed the qPCRs and prepared the sequencing libraries. AT collaborated with UI on the bioinformatics and statistical analysis and UI wrote the scripts to generate the raw figures. AT finalised figures and contributed to application of ecological theory. Results were interpreted by PM, AT, CN, TM, VO’F, and UI. AT drafted the manuscript. UI, FA, and VO’ revised the document. All authors approved the manuscript and agreed for accountability of the work therein.

## Conflict of Interest

The authors declare that the research was conducted in the absence of any commercial or financial relationships that could be construed as a potential conflict of interest.

## Footnotes

1 https://github.com/umerijaz/tutorials/blob/master/qiime2_tutorial.md

2 https://docs.qiime2.org/2021.2/tutorials/filtering/

3 https://github.com/gavinmdouglas/q2-picrust2
