# Supplementary material for: Combined Stochastic and Deterministic Processes Drive Community Assembly of Anaerobic Microbiomes During Granule Flotation
Source: Front Microbiol. 2021 May 14;12:666584. doi: 10.3389/fmicb.2021.666584 (PMC8160314; doi:10.3389/fmicb.2021.666584)
Supplement: Supplementary file 1 [file Data_Sheet_1.docx]

SUPPLEMENTAL MATERIAL

**COMBINED STOCHASTIC AND DETERMINISTIC PROCESSES DRIVE COMMUNITY ASSEMBLY OF ANAEROBIC MICROBIOMES DURING GRANULE FLOTATION**

Trego et al. 2021

SUPPLEMENTAL METHODS

*Long chain fatty acid (LCFA) concentration*

Calibration curves were produced from a series of standard solutions prepared for the following fatty acids: myristic (C14:0); palmitic (C16:0); palmitoleic (C16:1); stearic (C18:0); oleic (C18:1); and linoleic (C18:2). Each standard was prepared in a dichloromethane (DCM) solution using pentadecanoic (C15:0) acid as an internal standard (IS). 1.5-mL aliquots of the IS (1000 mg L^-1^) and HCl:1-propanol (25% v/v) were added to 2 mL of each standard. Separately, a 2-ml aliquot DCM, 1.5 mL of IS (1,000 mg L^-1^) and 1.5 ml of HCl:1-propanol (25% v/v) were added to 100 mg of dried biomass (*n*=3). Both standards and biomass-derived mixtures were vortex-mixed and digested at 100°C for 3.5 h. Following digestion, solutions were mixed with 2 mL ultra-pure water. Contact between the aqueous and organic phases was encouraged by shaking before the mixture rested for 30 min. The organic phase was collected the LCFA concentration was determined using a Varian Saturn 2000 GC/MS system (Varian Inc., Walnut Creek, CA).

*Statistical analyses*

The vegan package (Oksanen et al., 2015) was used for alpha and beta diversity analyses. For alpha diversity measures we used: **(i)** *rarefied richness* – the estimated number of species/features in a rarefied sample (to minimum library size); **(ii)** *Shannon entropy* – a commonly used index to measure balance within a community; **(iii)** *Simpson index –* a measure of dominance that weighs towards the abundance of the most common ASVs and is less sensitive to rare(r) ASVs; **(iv)** *Pilou eveness,* which compares the actual diversity values to the maximum possible diversity value, and is constrained between 0 and 1.0, whereby lower values will indicate more variation in abundance between different ASVs in the community; and **(v)** *Fisher’s alpha* – a parametric index of diversity that assumes the abundance of ASVs following the log series distribution. Ordination of ASV table in reduced space (beta diversity) was done using Principal Coordinate Analysis (PCoA) plots of ASVs using two different distance measures in Vegan’s cmdscale() function: **(1)** *Bray-Curtis,* which is a distance metric that considers only ASV abundance counts; and **(2)** *Weighted Unifrac,* which is a phylogenetic distance metric combining phylogenetic distance with relative abundances. Unifrac distances were calculated using the phyloseq package (McMurdie and Holmes, 2013).

To understand multivariate homogeneity of group dispersions (variances) between multiple conditions, Vegan's betadisper() function was used, in which the distances between objects and group centroids were handled by reducing the original distances (BrayCurtis, Unweighted Unifrac, or Weighted Unifrac) to principal coordinates and then performing ANOVA on them. Analysis of variance was performed using Vegan’s Adonis() against distance matrices (Bray-Curtis/Unweighted Unifrac/Weighted Unifrac). This function, referred to as PERMANOVA, fits linear models to distance matrices and used a permutation test with pseudo-F ratios.

To find ASVs/KEGG enzymes/MetaCyc pathways that are significantly different between different categories, we used DESeqDataSetFromMatrix() function from DESeq2 (Love et al., 2014) package with the adjusted p-value significance cut-off of 0.05 and log2 fold change cut-off of 2. This function uses negative binomial GLM to obtain maximum likelihood estimates for ASVs log fold change between two conditions. Then Bayesian shrinkage is applied to obtain shrunken log fold changes subsequently employing the Wald test for obtaining significances. For KEGG orthologs that were at least log2 fold significant, we used iPath3 (Darzi et al., 2018) to give an overview of KEGG pathways for microbial metabolic function.

Differential heat trees (Foster et al., 2017) were used to visualise differentially expressed taxa (using Wilcoxon p-value test adjusted with multiple comparison on the proportional microbiome data as recommended by the authors) comparing different categories.

We performed subset regression against different microbiome metrics by testing all possible combination of the explanatory variables, and then selecting the best model according to some statistical criteria, with recommendations given in (Kassambara, 2018) and code available at <http://www.sthda.com/english/articles/37-model-selection-essentials-in-r/155-best-subsets-regression-essentials-in-r/>. The R function regubsets() from leaps (Lumley and Miller, 2009) package was used to identify different best models of different sizes, by specifying the option nvmax, set to the maximum number of predictors to incorporate the model. Having obtained the best possible subsets, the k-fold cross-validation consisting of first dividing the data into k subsets. Each subset (10%) served successively as test data set and the remaining subset (90%) as training data. The average cross-validation error is then computed as the model prediction error. This was all done using a custom function utilising R’s train() function from the caret package (Kuhn, 2008). Finally R’s tab_model() function from sjPlot package (Lüdecke, 2018) was used to obtain the statistics for each model. To find the core microbiome, we have used R’s microbiome package (Lahti et al., 2017) and the prevalence of 85% of ASVs to be tagged as core microbiome, by following recommendations given in (Shetty et al., 2017).

For subset analysis, the “BVSTEP” routine (Clarke and Ainsworth, 1993) was used to search for the highest correlation, in a Mantel test, between dissimilarities of fixed and multivariate datasets. Briefly, the method calculates the Bray-Curtis distance between samples using all the ASVs and records it as original distances. It then permutes through the subset of ASVs, and for each permutation, it calculates the Bray-Curtis distances between the samples again, and correlates these distances against the original recorded distances until subsets are obtained that explain roughly the same beta diversity as the full set of ASVs. To run this algorithm, bvStep() (from the sinkr package) was used (Taylor, 2017). It permuted through 2^n^-1 possible combinations of features (n=1,829 ASVs) in the variable dataset.

SAMPLE SEQUENCE FILES

**Table S1.** Sequence file classifications

| **Name given in SRA** | **DNA vs. cDNA** | **Reactor** | **Category (floating vs. settled)** |
| --- | --- | --- | --- |
| cDNA_Top1 | cDNA | R1 | Floating Sludge Microbiome |
| cDNA_Top2 | cDNA | R1 | Floating Sludge Microbiome |
| cDNA_Top3 | cDNA | R2 | Floating Sludge Microbiome |
| cDNA_Top4 | cDNA | R2 | Floating Sludge Microbiome |
| cDNA_Top5 | cDNA | R3 | Floating Sludge Microbiome |
| cDNA_Top6 | cDNA | R3 | Floating Sludge Microbiome |
| cDNA_Bottom1 | cDNA | R1 | Settled Sludge Microbiome |
| cDNA_Bottom2 | cDNA | R1 | Settled Sludge Microbiome |
| cDNA_Bottom3 | cDNA | R2 | Settled Sludge Microbiome |
| cDNA_Bottom4 | cDNA | R2 | Settled Sludge Microbiome |
| cDNA_Bottom5 | cDNA | R3 | Settled Sludge Microbiome |
| cDNA_Bottom6 | cDNA | R3 | Settled Sludge Microbiome |
| DNA_Top1 | DNA | R1 | Floating Sludge Microbiome |
| DNA_Top2 | DNA | R1 | Floating Sludge Microbiome |
| DNA_Top3 | DNA | R2 | Floating Sludge Microbiome |
| DNA_Top4 | DNA | R2 | Floating Sludge Microbiome |
| DNA_Top5 | DNA | R3 | Floating Sludge Microbiome |
| DNA_Top6 | DNA | R3 | Floating Sludge Microbiome |
| DNA_Bottom1 | DNA | R1 | Settled Sludge Microbiome |
| DNA_Bottom2 | DNA | R1 | Settled Sludge Microbiome |
| DNA_Bottom3 | DNA | R2 | Settled Sludge Microbiome |
| DNA_Bottom4 | DNA | R2 | Settled Sludge Microbiome |
| DNA_Bottom5 | DNA | R3 | Settled Sludge Microbiome |
| DNA_Bottom6 | DNA | R3 | Settled Sludge Microbiome |

SUPPLEMENTAL FIGURES

**List of Supplemental Figures**

Figure S1. Picture of Bioreactors

Figure S2. Average Diameter

Figure S3. Alpha Diversity Measures

Figure S4. Unweighted UniFrac

Figure S5. Core Microbiome

Figure S6. Regression Heatmap

Figure S7. KOs and Pathways Diversity

Figure S8. DNA-based Differential Pathways

Figure S9. KO map of Functions

Figure S10. Stochasticity Ratio

Figure S11. Family Level Lottery Winners – Diversity and Prevalence

Figure S12. Genus Level Lottery Winners – Diversity and Prevalence


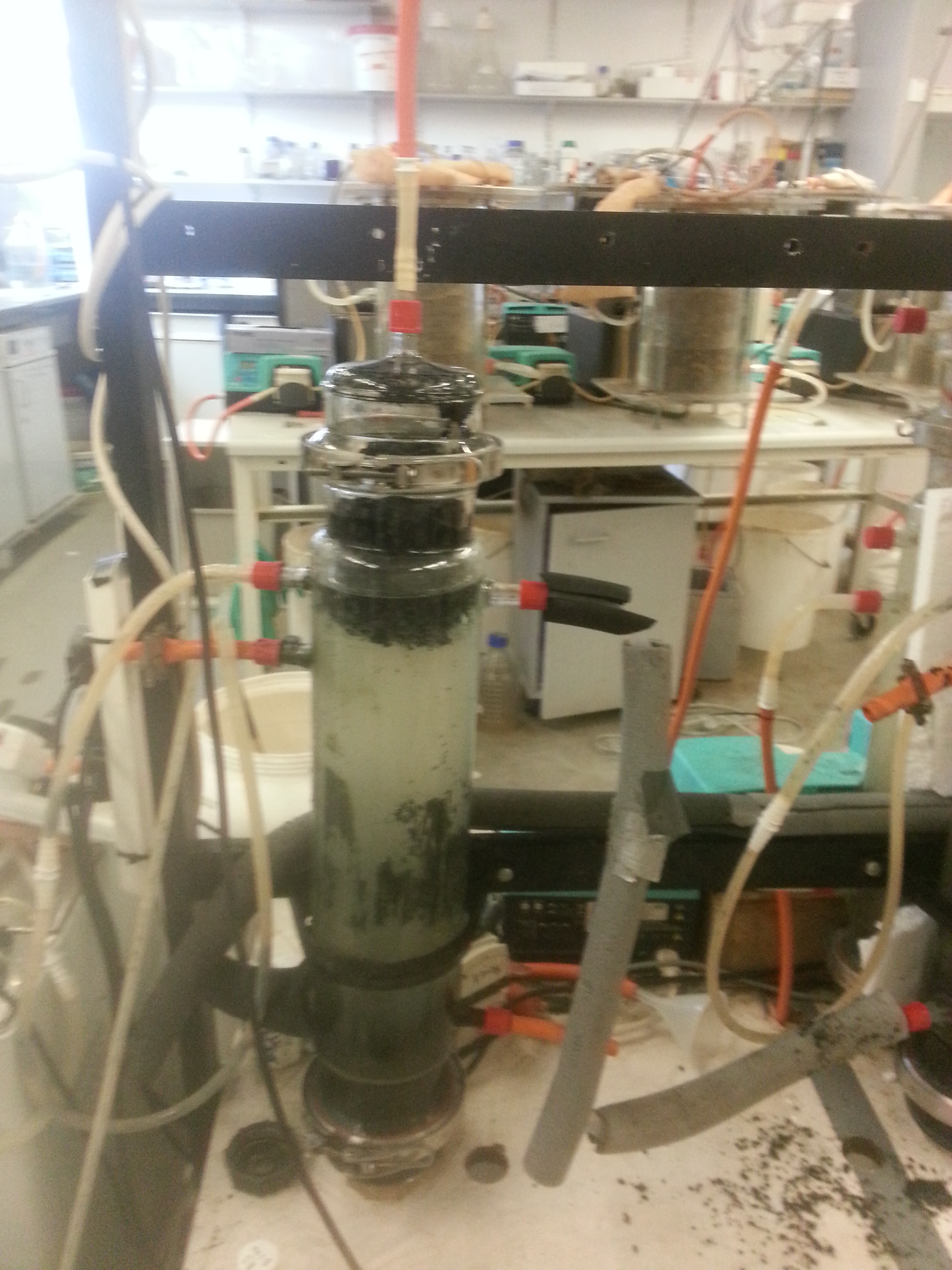


**Figure S1.** Photograph of reactor set-up with floating and settled sludge visible.


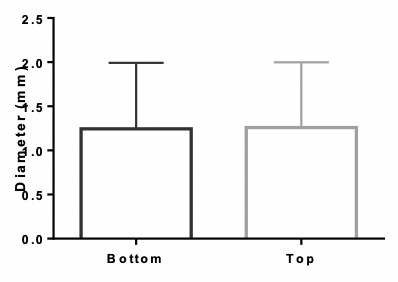


**Figure S2**. Average diameter (mm) of granules from both floating (Top) and settled (Bottom) biomass (*n*=341, per category).

**Figure S3.** Alpha diversity measures according to variances in the 16S rRNA genes from DNA and cDNA of floating (Top) and settled (Bottom) biomass determined using Fisher alpha, Pielou’s evenness, rareified Richness, Shannon entropy, and Simpson index. Samples are indicated by colour and lines connect two categories where the differences were significant (ANOVA) with * (p < 0.05), ** (p < 0.01), or *** (p < 0.001).

**Figure S4**. Microbial community diversity according to variances in 16S rRNA genes from both DNA and cDNA (*n=6*) measured using principle coordinate analysis (PCoA) with unweighted UniFrac distances. Samples are indicated by colour, and the ellipses are drawn at a 95% CI for all samples from each category, where arrows mark the direction of change in the community structure from mean ordination of settled samples to the mean ordination of floating samples for each nucleic acid type – the length indicating the amount of change.

**Figure S5.** Core community members, at 85% prevalence, from **(a)** DNA and **(b)** cDNA, where the y-axis represents the detection threshold at different abundances; note that the ASVs are sorted based on increasing abundance. In our study we first defined the core microbiome as genera that were present in 85% of the samples, an adjustable value but based previous recommendations(Jalanka-Tuovinen et al., 2011). Next, we applied several detection thresholds (essentially sequencing read counts) in terms of abundance to identify and sort low-abundant core groups from high-abundant core groups. The lower the detection threshold, the more groups are included in the core microbiome. At detection thresholds of 1-10, all genera in the core microbiome are found at >75% prevalence.

**Figure S6.** Summary heatmap of beta coefficients returned from subset regression analysis between the diversity parameters and the available meta-data, where red indicates a positive influence and blue, negative, consistently selected by the models in the analysis; significance indicated both by ‘heat’ and using stars with * (p < 0.05), ** (p < 0.01), or *** (p < 0.001).

**Figure S7.** Functional diversity in floating and settled granules according to variances in the 16S rRNA genes from DNA and cDNA and determined using PiCrust2 algorithms; where *Diversity of KOs* is shown by **(a)** the principal coordinate analysis (PCoA) using Bray-Curtis dissimilarity; box plots of **(b)** richness and **(c)** Shannon Entropy; *Diversity of Available Pathways* is shown using boxplots of **(d)** richness and **(e)** Shannon Entropy, and **(f)** PCoA using Bray Curtis dissimilarity; finally, **(g)** shows boxplots of the differential pathways, which were significantly (ANOVA) up-regulated in either floating or settled granules according to cDNA analysis, where the colour of the pathway indicates up-regulation (less negative value). Samples are indicated by colour, and the ellipses are drawn at a 95% CI for all samples from each category, where arrows mark the direction of change in the community structure from mean ordination of settled samples to the mean ordination of floating samples for each nucleic acid type – the length indicating the amount of change. Lines for figures b, c, d, & e connect two categories where the differences were significant (ANOVA) with * (p < 0.05), ** (p < 0.01), or *** (p < 0.001).

**Figure S8.** Boxplots of the differential pathways, which were significantly (ANOVA) up-regulated in either floating (Top) or settled (Bottom) granules according to DNA analysis.

**Figure S9.** Metabolic pathway map highlighting significantly upregulated in floating (green) and settled (red) granules determined using KOs. The lines represent individual (or groups of KOs). Highlighted ones were differentially enriched in either floating or settled biomass.


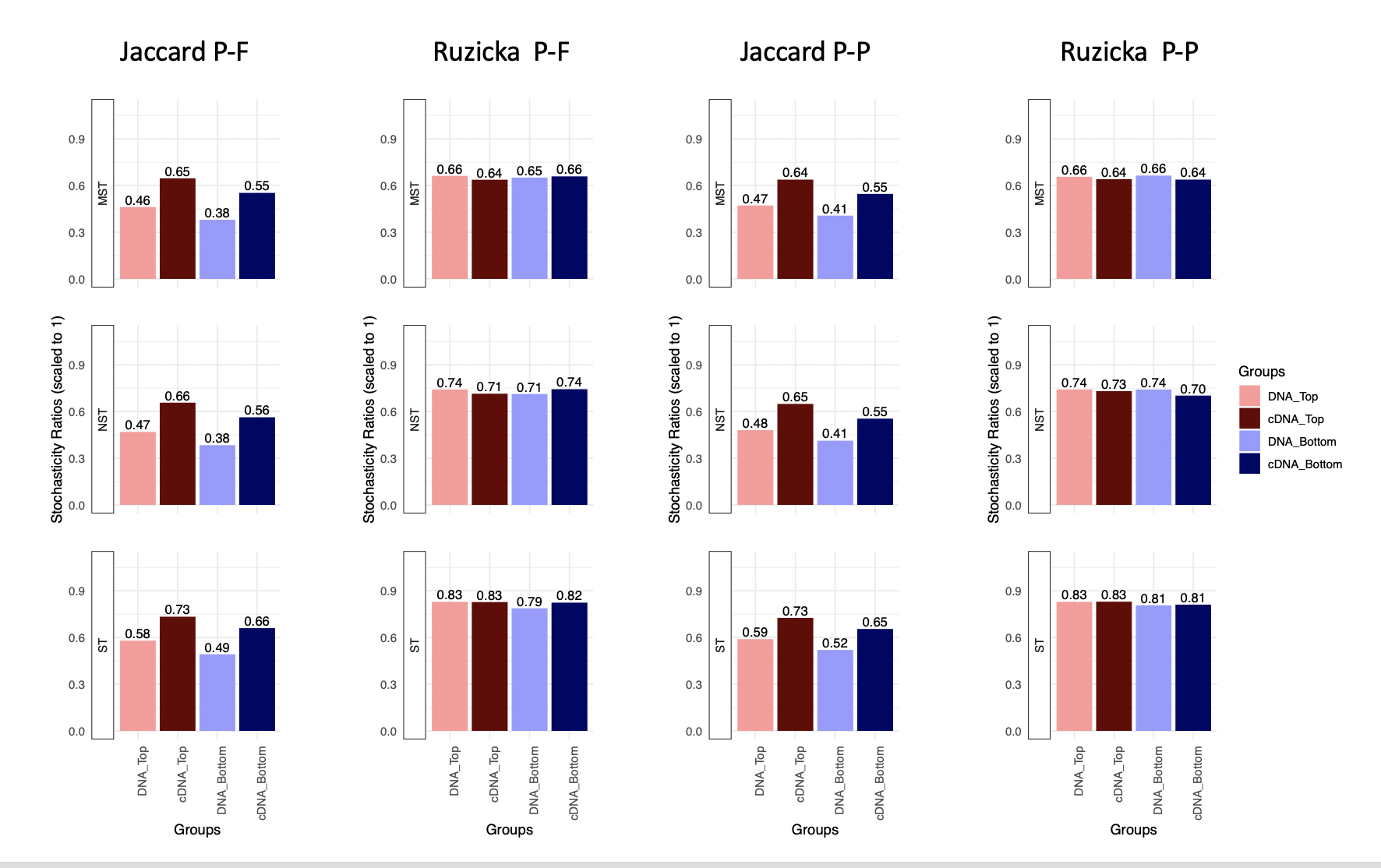


**Figure S10.** The stochasticity ratio approach, which quantified stochasticity as a stochasticity ratio (ST), normalised stochasticity ratio (NST) and a modified stochasticity ratio (MST) using the Jaccard (incidence-based) and Ružička (abundance-based) metrics as recommended by the authors (Ning et al., 2019) and Taxa-Richness constraints of proportional-proportional (P-P) and proportional-fixed (P-F).

**Figure S11.** Competitive lottery model for clade-based assembly which identified ‘winning’ ASVs (ASVs with >90% abundance within their defined clade) from families displaying lottery-like behaviour plotted according to the prevalence (the fraction of samples containing a winner within that family) and diversity (the frequency at which each ASV is selected as the winner from samples in which a winner was selected); the winners are identified on the heat tree.

**Figure S12.** Competitive lottery model for clade-based assembly which identified ‘winning’ ASVs (ASVs with >90% abundance within their defined clade) from genera displaying lottery-like behaviour plotted according to the prevalence (the fraction of samples containing a winner within that family) and diversity (the frequency at which each ASV is selected as the winner from samples in which a winner was selected); the winners are identified on the heat tree.

SUBSET REGRESSION ANALYSIS (Pages 20-37)

Richness Subset Regression

**Explanatory variables considered in the model are given below:**

| **Parameter** | **Description** |
| --- | --- |
| Status_cDNA | Nucleic acid type |
| Status_DNA | Nucleic acid type |
| Status_Floating | Floating biomass |
| Status_Settled | Settled biomass |
| Diameter | Average diameter of granules (mm) of floating vs settled biomass |
| Settling_Velocity | Average settling velocity (m h^-1^) of floating vs settled biomass |
| Percent_VS | Volatile solids concentration (%) of floating vs settled biomass |
| Total_LCFA | Long chain fatty acid concentration (mm gTS^-1^) of floating vs. settled biomass |
| Myristic_Acid | Acid concentration (mg gTS^-1^) of floating vs settled biomass |
| Palmitic_Acid | Acid concentration (mg gTS^-1^) of floating vs settled biomass |
| Stearic_Acid | Acid concentration (mg gTS^-1^) of floating vs settled biomass |
| Oleic_Acid | Acid concentration (mg gTS^-1^) of floating vs settled biomass |
| Linoleic_Acid | Acid concentration (mg gTS^-1^) of floating vs settled biomass |
| Bacterial_qPCR | Bacterial gene copy numbers (g_biomass_^-1^) of floating vs settled biomass |
| Archaea_qPCR | Archaeal gene copy numbers (g_biomass_^-1^) of floating vs settled biomass |

**Top models for Richness (Model parameters given afterwards with significant positive influencers highlighted in orange and negative in blue):**

| **Model** | **Cross validation errors** |
| --- | --- |
| M2 – Status_cDNA + Status_Floating | 48.13469 |
| M1 – Status_cDNA | 48.55886 |
| M3 – Status_DNA + Percent_VS + Bacterial+qPCR | 50.26512 |

|  | **Richness M1** | | | | | | | |
| --- | --- | --- | --- | --- | --- | --- | --- | --- |
| *Predictors* | *Estimates* | *std. Error* | *std. Beta* | *CI* | *standardized CI* | *Statistic* | *p* | *df* |
| (Intercept) | 248.15777 ^***^ | 14.09369 | -0.00000 | 218.92925 – 277.38629 | -0.38176 – 0.38176 | -0.00000 | **<0.001** | 22.00000 |
| Status_cDNA | 49.95300 ^*^ | 19.93148 | 0.47127 | 8.61764 – 91.28837 | 0.08130 – 0.86125 | 2.50624 | **0.020** | 22.00000 |
| Observations | 24 | | | | | | | |
| R^2^ / R^2^ adjusted | 0.222 / 0.187 | | | | | | | |
| ** p<0.05   ** p<0.01   *** p<0.001* | | | | | | | | |

|  | **Richness – M2** | | | | | | | |
| --- | --- | --- | --- | --- | --- | --- | --- | --- |
| *Predictors* | *Estimates* | *std. Error* | *std. Beta* | *CI* | *standardized CI* | *Statistic* | *p* | *df* |
| (Intercept) | 233.85276 ^***^ | 16.81971 | -0.00000 | 198.87425 – 268.83126 | -0.37303 – 0.37303 | -0.00000 | **<0.001** | 21.00000 |
| Status_cDNA | 49.95300 ^*^ | 19.42173 | 0.47127 | 9.56330 – 90.34270 | 0.09022 – 0.85232 | 2.57202 | **0.018** | 21.00000 |
| Status_Floating | 28.61002 | 19.42173 | 0.26992 | -11.77968 – 68.99972 | -0.11113 – 0.65097 | 1.47309 | 0.156 | 21.00000 |
| Observations | 24 | | | | | | | |
| R^2^ / R^2^ adjusted | 0.295 / 0.228 | | | | | | | |
| ** p<0.05   ** p<0.01   *** p<0.001* | | | | | | | | |

|  | **Richness – M3** | | | | | | | |
| --- | --- | --- | --- | --- | --- | --- | --- | --- |
| *Predictors* | *Estimates* | *std. Error* | *std. Beta* | *CI* | *standardized CI* | *Statistic* | *p* | *df* |
| (Intercept) | -92.63329 | 417.85057 | 0.00000 | -964.25431 – 778.98772 | -0.37163 – 0.37163 | 0.00000 | 0.827 | 20.00000 |
| Status_DNA | -58.74036 ^*^ | 20.78637 | -0.55418 | -102.09997 – -15.38075 | -0.96325 – -0.14511 | -2.82591 | **0.010** | 20.00000 |
| Percent_VS | 131.33648 | 71.44136 | 0.49563 | -17.68758 – 280.36054 | -0.06675 – 1.05801 | 1.83838 | 0.081 | 20.00000 |
| Bacterial_qPCR | -66.74730 | 58.82087 | -0.31696 | -189.44549 – 55.95090 | -0.89962 – 0.26570 | -1.13476 | 0.270 | 20.00000 |
| Observations | 24 | | | | | | | |
| R^2^ / R^2^ adjusted | 0.338 / 0.238 | | | | | | | |
| ** p<0.05   ** p<0.01   *** p<0.001* | | | | | | | | |

Shannon Subset Regression

**Explanatory variables considered in the model are given below:**

| **Parameter** | **Description** |
| --- | --- |
| Status_cDNA | Nucleic acid type |
| Status_DNA | Nucleic acid type |
| Status_Floating | Floating biomass |
| Status_Settled | Settled biomass |
| Diameter | Average diameter of granules (mm) of floating vs settled biomass |
| Settling_Velocity | Average settling velocity (m h^-1^) of floating vs settled biomass |
| Percent_VS | Volatile solids concentration (%) of floating vs settled biomass |
| Total_LCFA | Long chain fatty acid concentration (mm gTS^-1^) of floating vs. settled biomass |
| Myristic_Acid | Acid concentration (mg gTS^-1^) of floating vs settled biomass |
| Palmitic_Acid | Acid concentration (mg gTS^-1^) of floating vs settled biomass |
| Stearic_Acid | Acid concentration (mg gTS^-1^) of floating vs settled biomass |
| Oleic_Acid | Acid concentration (mg gTS^-1^) of floating vs settled biomass |
| Linoleic_Acid | Acid concentration (mg gTS^-1^) of floating vs settled biomass |
| Bacterial_qPCR | Bacterial gene copy numbers (g_biomass_^-1^) of floating vs settled biomass |
| Archaea_qPCR | Archaeal gene copy numbers (g_biomass_^-1^) of floating vs settled biomass |

**Top models for Shannon (Model parameters given afterwards with significant positive influencers highlighted in orange and negative in blue):**

| **Model** | **Cross validation errors** |
| --- | --- |
| M2 – Status_cDNA + Status_Floating | 0.24462 |
| M3 – Status_DNA + Palmitic_Acid + Bacterial_qPCR | 0.24591 |
| M1 – Status_Floating | 0.24784 |

|  | **Shannon M1** | | | | | | | |
| --- | --- | --- | --- | --- | --- | --- | --- | --- |
| *Predictors* | *Estimates* | *std. Error* | *std. Beta* | *CI* | *standardized CI* | *Statistic* | *p* | *df* |
| (Intercept) | 3.73354 ^***^ | 0.07444 | -0.00000 | 3.57916 – 3.88791 | -0.30688 – 0.30688 | -0.00000 | **<0.001** | 22.00000 |
| Status_Floating | 0.49115 ^***^ | 0.10527 | 0.70523 | 0.27283 – 0.70947 | 0.39175 – 1.01870 | 4.66556 | **<0.001** | 22.00000 |
| Observations | 24 | | | | | | | |
| R^2^ / R^2^ adjusted | 0.497 / 0.474 | | | | | | | |
| ** p<0.05   ** p<0.01   *** p<0.001* | | | | | | | | |

|  | **Shannon – M2** | | | | | | | |
| --- | --- | --- | --- | --- | --- | --- | --- | --- |
| *Predictors* | *Estimates* | *std. Error* | *std. Beta* | *CI* | *standardized CI* | *Statistic* | *p* | *df* |
| (Intercept) | 3.84957 ^***^ | 0.08236 | -0.00000 | 3.67829 – 4.02086 | -0.27801 – 0.27801 | -0.00000 | **<0.001** | 21.00000 |
| Status_cDNA | -0.23207 ^*^ | 0.09511 | -0.33323 | -0.42986 – -0.03429 | -0.61722 – -0.04924 | -2.44019 | **0.024** | 21.00000 |
| Status_Floating | 0.49115 ^***^ | 0.09511 | 0.70523 | 0.29337 – 0.68893 | 0.42124 – 0.98922 | 5.16426 | **<0.001** | 21.00000 |
| Observations | 24 | | | | | | | |
| R^2^ / R^2^ adjusted | 0.608 / 0.571 | | | | | | | |
| ** p<0.05   ** p<0.01   *** p<0.001* | | | | | | | | |

|  | **Shannon – M3** | | | | | | | |
| --- | --- | --- | --- | --- | --- | --- | --- | --- |
| *Predictors* | *Estimates* | *std. Error* | *std. Beta* | *CI* | *standardized CI* | *Statistic* | *p* | *df* |
| (Intercept) | 3.61451 | 3.27413 | -0.00000 | -3.21520 – 10.44422 | -0.28348 – 0.28348 | -0.00000 | 0.283 | 20.00000 |
| Status_DNA | 0.25405 ^*^ | 0.10418 | 0.36478 | 0.03673 – 0.47137 | 0.05274 – 0.67683 | 2.43853 | **0.024** | 20.00000 |
| Palmitic_Acid | -0.05675 ^**^ | 0.01885 | -0.61932 | -0.09606 – -0.01744 | -1.04831 – -0.19033 | -3.01146 | **0.007** | 20.00000 |
| Bacterial_qPCR | 0.16692 | 0.29481 | 0.12064 | -0.44805 – 0.78188 | -0.32382 – 0.56510 | 0.56618 | 0.578 | 20.00000 |
| Observations | 24 | | | | | | | |
| R^2^ / R^2^ adjusted | 0.615 / 0.557 | | | | | | | |
| ** p<0.05   ** p<0.01   *** p<0.001* | | | | | | | | |

Simpson Subset Regression

**Explanatory variables considered in the model are given below:**

| **Parameter** | **Description** |
| --- | --- |
| Status_cDNA | Nucleic acid type |
| Status_DNA | Nucleic acid type |
| Status_Floating | Floating biomass |
| Status_Settled | Settled biomass |
| Diameter | Average diameter of granules (mm) of floating vs settled biomass |
| Settling_Velocity | Average settling velocity (m h^-1^) of floating vs settled biomass |
| Percent_VS | Volatile solids concentration (%) of floating vs settled biomass |
| Total_LCFA | Long chain fatty acid concentration (mm gTS^-1^) of floating vs. settled biomass |
| Myristic_Acid | Acid concentration (mg gTS^-1^) of floating vs settled biomass |
| Palmitic_Acid | Acid concentration (mg gTS^-1^) of floating vs settled biomass |
| Stearic_Acid | Acid concentration (mg gTS^-1^) of floating vs settled biomass |
| Oleic_Acid | Acid concentration (mg gTS^-1^) of floating vs settled biomass |
| Linoleic_Acid | Acid concentration (mg gTS^-1^) of floating vs settled biomass |
| Bacterial_qPCR | Bacterial gene copy numbers (g_biomass_^-1^) of floating vs settled biomass |
| Archaea_qPCR | Archaeal gene copy numbers (g_biomass_^-1^) of floating vs settled biomass |

**Top models for Simpson (Model parameters given afterwards with significant positive influencers highlighted in orange and negative in blue):**

| **Model** | **Cross validation errors** |
| --- | --- |
| M2 – Status_cDNA + Archaea_qPCR | 0.01466 |
| M3 – Status_cDNA + Status_Settled + Bacterial_qPCR | 0.01487 |
| M1 – Status_Floating | 0.02135 |

|  | **Simpson – M1** | | | | | | | |
| --- | --- | --- | --- | --- | --- | --- | --- | --- |
| *Predictors* | *Estimates* | *std. Error* | *std. Beta* | *CI* | *standardized CI* | *Statistic* | *p* | *df* |
| (Intercept) | 0.92329 ^***^ | 0.00657 | 0.00000 | 0.90967 – 0.93691 | -0.32585 – 0.32585 | 0.00000 | **<0.001** | 22.00000 |
| Status_Floating | 0.03809 ^***^ | 0.00929 | 0.65823 | 0.01883 – 0.05735 | 0.32537 – 0.99109 | 4.10107 | **<0.001** | 22.00000 |
| Observations | 24 | | | | | | | |
| R^2^ / R^2^ adjusted | 0.433 / 0.408 | | | | | | | |
| ** p<0.05   ** p<0.01   *** p<0.001* | | | | | | | | |

|  | **Simpson – M2** | | | | | | | |
| --- | --- | --- | --- | --- | --- | --- | --- | --- |
| *Predictors* | *Estimates* | *std. Error* | *std. Beta* | *CI* | *standardized CI* | *Statistic* | *p* | *df* |
| (Intercept) | 0.09106 | 0.12254 | 0.00000 | -0.16379 – 0.34590 | -0.19810 – 0.19810 | 0.00000 | 0.466 | 21.00000 |
| Status_cDNA | -0.07392 ^***^ | 0.00806 | -1.27745 | -0.09069 – -0.05715 | -1.56723 – -0.98767 | -9.16776 | **<0.001** | 21.00000 |
| Archaea_qPCR | 0.09199 ^***^ | 0.01298 | 0.98727 | 0.06499 – 0.11899 | 0.69750 – 1.27705 | 7.08528 | **<0.001** | 21.00000 |
| Observations | 24 | | | | | | | |
| R^2^ / R^2^ adjusted | 0.801 / 0.782 | | | | | | | |
| ** p<0.05   ** p<0.01   *** p<0.001* | | | | | | | | |

|  | **Simpson – M3** | | | | | | | |
| --- | --- | --- | --- | --- | --- | --- | --- | --- |
| *Predictors* | *Estimates* | *std. Error* | *std. Beta* | *CI* | *standardized CI* | *Statistic* | *p* | *df* |
| (Intercept) | 0.54791 ^**^ | 0.16672 | 0.00000 | 0.20014 – 0.89567 | -0.19413 – 0.19413 | 0.00000 | **0.004** | 20.00000 |
| Status_cDNA | -0.03873 ^***^ | 0.00593 | -0.66927 | -0.05109 – -0.02636 | -0.88296 – -0.45558 | -6.53310 | **<0.001** | 20.00000 |
| Status_Settled | -0.02257 ^*^ | 0.00815 | -0.39013 | -0.03957 – -0.00558 | -0.68390 – -0.09635 | -2.77009 | **0.012** | 20.00000 |
| Bacterial_qPCR | 0.04328 ^*^ | 0.01677 | 0.37649 | 0.00829 – 0.07827 | 0.07212 – 0.68086 | 2.58022 | **0.018** | 20.00000 |
| Observations | 24 | | | | | | | |
| R^2^ / R^2^ adjusted | 0.819 / 0.792 | | | | | | | |
| ** p<0.05   ** p<0.01   *** p<0.001* | | | | | | | | |

Fisher Alpha Subset Regression

**Explanatory variables considered in the model are given below:**

| **Parameter** | **Description** |
| --- | --- |
| Status_cDNA | Nucleic acid type |
| Status_DNA | Nucleic acid type |
| Status_Floating | Floating biomass |
| Status_Settled | Settled biomass |
| Diameter | Average diameter of granules (mm) of floating vs settled biomass |
| Settling_Velocity | Average settling velocity (m h^-1^) of floating vs settled biomass |
| Percent_VS | Volatile solids concentration (%) of floating vs settled biomass |
| Total_LCFA | Long chain fatty acid concentration (mm gTS^-1^) of floating vs. settled biomass |
| Myristic_Acid | Acid concentration (mg gTS^-1^) of floating vs settled biomass |
| Palmitic_Acid | Acid concentration (mg gTS^-1^) of floating vs settled biomass |
| Stearic_Acid | Acid concentration (mg gTS^-1^) of floating vs settled biomass |
| Oleic_Acid | Acid concentration (mg gTS^-1^) of floating vs settled biomass |
| Linoleic_Acid | Acid concentration (mg gTS^-1^) of floating vs settled biomass |
| Bacterial_qPCR | Bacterial gene copy numbers (g_biomass_^-1^) of floating vs settled biomass |
| Archaea_qPCR | Archaeal gene copy numbers (g_biomass_^-1^) of floating vs settled biomass |

**Top models (Model parameters given afterwards with significant positive influencers highlighted in orange and negative in blue):**

| **Model** | **Cross validation errors** |
| --- | --- |
| M2 – Status_cDNA + Status_Floating | 8.20297 |
| M1 – Status _DNA | 8.25045 |
| M3 – Status_cDNA + Status_Settled + Bacterial_qPCR | 8.58702 |

|  | **FisherAlpha M1** | | | | | | | |
| --- | --- | --- | --- | --- | --- | --- | --- | --- |
| *Predictors* | *Estimates* | *std. Error* | *std. Beta* | *CI* | *standardized CI* | *Statistic* | *p* | *df* |
| (Intercept) | 44.60566 ^***^ | 2.40611 | 0.00000 | 39.61569 – 49.59564 | -0.39400 – 0.39400 | 0.00000 | **<0.001** | 22.00000 |
| Status_DNA | -7.25974 ^*^ | 3.40276 | -0.41404 | -14.31663 – -0.20285 | -0.81651 – -0.01157 | -2.13349 | **0.044** | 22.00000 |
| Observations | 24 | | | | | | | |
| R^2^ / R^2^ adjusted | 0.171 / 0.134 | | | | | | | |
| ** p<0.05   ** p<0.01   *** p<0.001* | | | | | | | | |

|  | **FisherAlpha M2** | | | | | | | |
| --- | --- | --- | --- | --- | --- | --- | --- | --- |
| *Predictors* | *Estimates* | *std. Error* | *std. Beta* | *CI* | *standardized CI* | *Statistic* | *p* | *df* |
| (Intercept) | 34.89445 ^***^ | 2.87038 | 0.00000 | 28.92517 – 40.86372 | -0.38483 – 0.38483 | 0.00000 | **<0.001** | 21.00000 |
| Status_cDNA | 7.25974 ^*^ | 3.31443 | 0.41404 | 0.36701 – 14.15246 | 0.02093 – 0.80715 | 2.19035 | **0.040** | 21.00000 |
| Status_Floating | 4.90295 | 3.31443 | 0.27963 | -1.98977 – 11.79568 | -0.11348 – 0.67274 | 1.47928 | 0.154 | 21.00000 |
| Observations | 24 | | | | | | | |
| R^2^ / R^2^ adjusted | 0.250 / 0.178 | | | | | | | |
| ** p<0.05   ** p<0.01   *** p<0.001* | | | | | | | | |

|  | **FisherAlpha M3** | | | | | | | |
| --- | --- | --- | --- | --- | --- | --- | --- | --- |
| *Predictors* | *Estimates* | *std. Error* | *std. Beta* | *CI* | *standardized CI* | *Statistic* | *p* | *df* |
| (Intercept) | 149.40317 | 99.96627 | -0.00000 | -59.12281 – 357.92915 | -0.38415 – 0.38415 | -0.00000 | 0.151 | 20.00000 |
| Status_cDNA | 8.71218 ^*^ | 3.55437 | 0.49688 | 1.29788 – 16.12647 | 0.07402 – 0.91973 | 2.45111 | **0.024** | 20.00000 |
| Status_Settled | -8.85738 | 4.88646 | -0.50516 | -19.05035 – 1.33559 | -1.08649 – 0.07617 | -1.81264 | 0.085 | 20.00000 |
| Bacterial_qPCR | -11.03248 | 10.05810 | -0.31671 | -32.01331 – 9.94834 | -0.91901 – 0.28559 | -1.09688 | 0.286 | 20.00000 |
| Observations | 24 | | | | | | | |
| R^2^ / R^2^ adjusted | 0.292 / 0.186 | | | | | | | |
| ** p<0.05   ** p<0.01   *** p<0.001* | | | | | | | | |

Pielou Evenness Subset Regression

**Explanatory variables considered in the model are given below:**

| **Parameter** | **Description** |
| --- | --- |
| Status_cDNA | Nucleic acid type |
| Status_DNA | Nucleic acid type |
| Status_Floating | Floating biomass |
| Status_Settled | Settled biomass |
| Diameter | Average diameter of granules (mm) of floating vs settled biomass |
| Settling_Velocity | Average settling velocity (m h^-1^) of floating vs settled biomass |
| Percent_VS | Volatile solids concentration (%) of floating vs settled biomass |
| Total_LCFA | Long chain fatty acid concentration (mm gTS^-1^) of floating vs. settled biomass |
| Myristic_Acid | Acid concentration (mg gTS^-1^) of floating vs settled biomass |
| Palmitic_Acid | Acid concentration (mg gTS^-1^) of floating vs settled biomass |
| Stearic_Acid | Acid concentration (mg gTS^-1^) of floating vs settled biomass |
| Oleic_Acid | Acid concentration (mg gTS^-1^) of floating vs settled biomass |
| Linoleic_Acid | Acid concentration (mg gTS^-1^) of floating vs settled biomass |
| Bacterial_qPCR | Bacterial gene copy numbers (g_biomass_^-1^) of floating vs settled biomass |
| Archaea_qPCR | Archaeal gene copy numbers (g_biomass_^-1^) of floating vs settled biomass |

**Top models for Pielou Evenness (Model parameters given afterwards with significant positive influencers highlighted in orange and negative in blue):**

| **Model** | **Cross validation errors** |
| --- | --- |
| M2 – Status_cDNA + Archaea_qPCR | 0.02985 |
| M3 – Status_cDNA + Myristic_Acid + Bacterial+qPCR | 0.03011 |
| M1 – Status_Floating | 0.04288 |

|  | **PielouEvenness – M1** | | | | | | | |
| --- | --- | --- | --- | --- | --- | --- | --- | --- |
| *Predictors* | *Estimates* | *std. Error* | *std. Beta* | *CI* | *standardized CI* | *Statistic* | *p* | *df* |
| (Intercept) | 0.67529 ^***^ | 0.01330 | -0.00000 | 0.64770 – 0.70288 | -0.33734 – 0.33734 | -0.00000 | **<0.001** | 22.00000 |
| Status_Floating | 0.07096 ^**^ | 0.01882 | 0.62658 | 0.03193 – 0.10998 | 0.28199 – 0.97118 | 3.77100 | **0.001** | 22.00000 |
| Observations | 24 | | | | | | | |
| R^2^ / R^2^ adjusted | 0.393 / 0.365 | | | | | | | |
| ** p<0.05   ** p<0.01   *** p<0.001* | | | | | | | | |

|  | **PielouEvenness – M2** | | | | | | | |
| --- | --- | --- | --- | --- | --- | --- | --- | --- |
| *Predictors* | *Estimates* | *std. Error* | *std. Beta* | *CI* | *standardized CI* | *Statistic* | *p* | *df* |
| (Intercept) | -0.81868 ^**^ | 0.25675 | -0.00000 | -1.35262 – -0.28474 | -0.21209 – 0.21209 | -0.00000 | **0.004** | 21.00000 |
| Status_cDNA | -0.14248 ^***^ | 0.01689 | -1.25819 | -0.17761 – -0.10735 | -1.56842 – -0.94796 | -8.43414 | **<0.001** | 21.00000 |
| Archaea_qPCR | 0.16578 ^***^ | 0.02720 | 0.90912 | 0.10921 – 0.22235 | 0.59888 – 1.21935 | 6.09416 | **<0.001** | 21.00000 |
| Observations | 24 | | | | | | | |
| R^2^ / R^2^ adjusted | 0.772 / 0.750 | | | | | | | |
| ** p<0.05   ** p<0.01   *** p<0.001* | | | | | | | | |

|  | **PielouEvenness – M3** | | | | | | | |
| --- | --- | --- | --- | --- | --- | --- | --- | --- |
| *Predictors* | *Estimates* | *std. Error* | *std. Beta* | *CI* | *standardized CI* | *Statistic* | *p* | *df* |
| (Intercept) | 0.22015 | 0.37174 | -0.00000 | -0.55528 – 0.99559 | -0.20488 – 0.20488 | -0.00000 | 0.560 | 20.00000 |
| Status_cDNA | -0.07754 ^***^ | 0.01224 | -0.68473 | -0.10308 – -0.05200 | -0.91025 – -0.45921 | -6.33340 | **<0.001** | 20.00000 |
| Myristic_Acid | -0.01813 ^*^ | 0.00647 | -0.41625 | -0.03163 – -0.00463 | -0.72629 – -0.10620 | -2.80049 | **0.011** | 20.00000 |
| Bacterial_qPCR | 0.06645 | 0.03465 | 0.29538 | -0.00582 – 0.13872 | -0.02585 – 0.61660 | 1.91811 | 0.069 | 20.00000 |
| Observations | 24 | | | | | | | |
| R^2^ / R^2^ adjusted | 0.799 / 0.768 | | | | | | | |
| ** p<0.05   ** p<0.01   *** p<0.001* | | | | | | | | |

Environmental Filtering Subset Regression

**Explanatory variables considered in the model are given below:**

| **Parameter** | **Description** |
| --- | --- |
| Status_cDNA | Nucleic acid type |
| Status_DNA | Nucleic acid type |
| Status_Floating | Floating biomass |
| Status_Settled | Settled biomass |
| Diameter | Average diameter of granules (mm) of floating vs settled biomass |
| Settling_Velocity | Average settling velocity (m h^-1^) of floating vs settled biomass |
| Percent_VS | Volatile solids concentration (%) of floating vs settled biomass |
| Total_LCFA | Long chain fatty acid concentration (mm gTS^-1^) of floating vs. settled biomass |
| Myristic_Acid | Acid concentration (mg gTS^-1^) of floating vs settled biomass |
| Palmitic_Acid | Acid concentration (mg gTS^-1^) of floating vs settled biomass |
| Stearic_Acid | Acid concentration (mg gTS^-1^) of floating vs settled biomass |
| Oleic_Acid | Acid concentration (mg gTS^-1^) of floating vs settled biomass |
| Linoleic_Acid | Acid concentration (mg gTS^-1^) of floating vs settled biomass |
| Bacterial_qPCR | Bacterial gene copy numbers (g_biomass_^-1^) of floating vs settled biomass |
| Archaea_qPCR | Archaeal gene copy numbers (g_biomass_^-1^) of floating vs settled biomass |

**Top models for Environmental Filtering using NRI and NTI (Model parameters given afterwards with significant positive influencers highlighted in orange and negative in blue):**

| **Model** | **Cross validation errors** |
| --- | --- |
| M2 – Status_cDNA + Status_Floating | 0.72074 |
| M3 – Status_cDNA + Status_Floating + Bacterial+qPCR | 0.75934 |
| M1 – Status_Settled | 0.77784 |

|  | **NRI – M1** | | | | | | | |
| --- | --- | --- | --- | --- | --- | --- | --- | --- |
| *Predictors* | *Estimates* | *std. Error* | *std. Beta* | *CI* | *standardized CI* | *Statistic* | *p* | *df* |
| (Intercept) | 1.50523 ^***^ | 0.21399 | 0.00000 | 1.06145 – 1.94902 | -0.38993 – 0.38993 | 0.00000 | **<0.001** | 22.00000 |
| Status_Settled | 0.68397 ^*^ | 0.30263 | 0.43409 | 0.05636 – 1.31158 | 0.03577 – 0.83241 | 2.26013 | **0.034** | 22.00000 |
| Observations | 24 | | | | | | | |
| R^2^ / R^2^ adjusted | 0.188 / 0.152 | | | | | | | |
| ** p<0.05   ** p<0.01   *** p<0.001* | | | | | | | | |

|  | **NRI – M2** | | | | | | | |
| --- | --- | --- | --- | --- | --- | --- | --- | --- |
| *Predictors* | *Estimates* | *std. Error* | *std. Beta* | *CI* | *standardized CI* | *Statistic* | *p* | *df* |
| (Intercept) | 1.92358 ^***^ | 0.24875 | 0.00000 | 1.40627 – 2.44089 | -0.37113 – 0.37113 | 0.00000 | **<0.001** | 21.00000 |
| Status_cDNA | 0.53125 | 0.28724 | 0.33717 | -0.06608 – 1.12859 | -0.04194 – 0.71628 | 1.84954 | 0.079 | 21.00000 |
| Status_Floating | -0.68397 ^*^ | 0.28724 | -0.43409 | -1.28131 – -0.08663 | -0.81320 – -0.05498 | -2.38123 | **0.027** | 21.00000 |
| Observations | 24 | | | | | | | |
| R^2^ / R^2^ adjusted | 0.302 / 0.236 | | | | | | | |
| ** p<0.05   ** p<0.01   *** p<0.001* | | | | | | | | |

|  | **NRI – M3** | | | | | | | |
| --- | --- | --- | --- | --- | --- | --- | --- | --- |
| *Predictors* | *Estimates* | *std. Error* | *std. Beta* | *CI* | *standardized CI* | *Statistic* | *p* | *df* |
| (Intercept) | 3.77914 | 8.58849 | -0.00000 | -14.13615 – 21.69442 | -0.38101 – 0.38101 | -0.00000 | 0.665 | 20.00000 |
| Status_cDNA | 0.55676 | 0.31679 | 0.35336 | -0.10405 – 1.21758 | -0.06604 – 0.77275 | 1.75752 | 0.094 | 20.00000 |
| Status_Floating | -0.61452 | 0.43551 | -0.39001 | -1.52299 – 0.29394 | -0.96658 – 0.18656 | -1.41103 | 0.174 | 20.00000 |
| Bacterial_qPCR | -0.19376 | 0.89645 | -0.06190 | -2.06372 – 1.67619 | -0.65926 – 0.53547 | -0.21615 | 0.831 | 20.00000 |
| Observations | 24 | | | | | | | |
| R^2^ / R^2^ adjusted | 0.304 / 0.199 | | | | | | | |
| ** p<0.05   ** p<0.01   *** p<0.001* | | | | | | | | |

LCBD Subset Regression

**Explanatory variables considered in the model are given below:**

| **Parameter** | **Description** |
| --- | --- |
| Status_cDNA | Nucleic acid type |
| Status_DNA | Nucleic acid type |
| Status_Floating | Floating biomass |
| Status_Settled | Settled biomass |
| Diameter | Average diameter of granules (mm) of floating vs settled biomass |
| Settling_Velocity | Average settling velocity (m h^-1^) of floating vs settled biomass |
| Percent_VS | Volatile solids concentration (%) of floating vs settled biomass |
| Total_LCFA | Long chain fatty acid concentration (mm gTS^-1^) of floating vs. settled biomass |
| Myristic_Acid | Acid concentration (mg gTS^-1^) of floating vs settled biomass |
| Palmitic_Acid | Acid concentration (mg gTS^-1^) of floating vs settled biomass |
| Stearic_Acid | Acid concentration (mg gTS^-1^) of floating vs settled biomass |
| Oleic_Acid | Acid concentration (mg gTS^-1^) of floating vs settled biomass |
| Linoleic_Acid | Acid concentration (mg gTS^-1^) of floating vs settled biomass |
| Bacterial_qPCR | Bacterial gene copy numbers (g_biomass_^-1^) of floating vs settled biomass |
| Archaea_qPCR | Archaeal gene copy numbers (g_biomass_^-1^) of floating vs settled biomass |

**Top models for LCBD using BRAY CURTIS DISTANCES (Model parameters given afterwards with significant positive influencers highlighted in orange and negative in blue):**

| **Model** | **Cross validation errors** |
| --- | --- |
| M1 – Bacterial_qPCR | 0.00535 |
| M2 – Bacterial_qPCR + Archaea_qPCR | 0.00553 |
| M3 – Status_cDNA + Status_Floating + Bacterial_qPCR | 0.00569 |

|  | **LCBD – Bray Curtis – M1** | | | | | | | |
| --- | --- | --- | --- | --- | --- | --- | --- | --- |
| *Predictors* | *Estimates* | *std. Error* | *std. Beta* | *CI* | *standardized CI* | *Statistic* | *p* | *df* |
| (Intercept) | 0.01544 | 0.04282 | 0.00000 | -0.07336 – 0.10424 | -0.42920 – 0.42920 | 0.00000 | 0.722 | 22.00000 |
| Bacterial_qPCR | 0.00267 | 0.00436 | 0.12953 | -0.00637 – 0.01171 | -0.30890 – 0.56795 | 0.61270 | 0.546 | 22.00000 |
| Observations | 24 | | | | | | | |
| R^2^ / R^2^ adjusted | 0.017 / -0.028 | | | | | | | |
| ** p<0.05   ** p<0.01   *** p<0.001* | | | | | | | | |

|  | **LCBD – Bray Curtis – M2** | | | | | | | |
| --- | --- | --- | --- | --- | --- | --- | --- | --- |
| *Predictors* | *Estimates* | *std. Error* | *std. Beta* | *CI* | *standardized CI* | *Statistic* | *p* | *df* |
| (Intercept) | 0.01377 | 0.04388 | 0.00000 | -0.07749 – 0.10502 | -0.43893 – 0.43893 | 0.00000 | 0.757 | 21.00000 |
| Bacterial_qPCR | 0.00545 | 0.00841 | 0.26416 | -0.01204 – 0.02293 | -0.58415 – 1.11247 | 0.64759 | 0.524 | 21.00000 |
| Archaea_qPCR | -0.00265 | 0.00682 | -0.15860 | -0.01682 – 0.01152 | -1.00691 – 0.68971 | -0.38880 | 0.701 | 21.00000 |
| Observations | 24 | | | | | | | |
| R^2^ / R^2^ adjusted | 0.024 / -0.069 | | | | | | | |
| ** p<0.05   ** p<0.01   *** p<0.001* | | | | | | | | |

|  | **LCBD – Bray Curtis – M3** | | | | | | | |
| --- | --- | --- | --- | --- | --- | --- | --- | --- |
| *Predictors* | *Estimates* | *std. Error* | *std. Beta* | *CI* | *standardized CI* | *Statistic* | *p* | *df* |
| (Intercept) | 0.00555 | 0.06697 | 0.00000 | -0.13416 – 0.14525 | -0.45114 – 0.45114 | 0.00000 | 0.935 | 20.00000 |
| Status_cDNA | -0.00094 | 0.00247 | -0.09046 | -0.00609 – 0.00421 | -0.58705 – 0.40613 | -0.38000 | 0.708 | 20.00000 |
| Status_Floating | -0.00042 | 0.00340 | -0.04010 | -0.00750 – 0.00667 | -0.72279 – 0.64260 | -0.12251 | 0.904 | 20.00000 |
| Bacterial_qPCR | 0.00375 | 0.00699 | 0.18174 | -0.01084 – 0.01833 | -0.52558 – 0.88906 | 0.53597 | 0.598 | 20.00000 |
| Observations | 24 | | | | | | | |
| R^2^ / R^2^ adjusted | 0.024 / -0.123 | | | | | | | |
| ** p<0.05   ** p<0.01   *** p<0.001* | | | | | | | | |

**Top models for LCBD using UniFrac DISTANCES (Model parameters given afterwards with significant positive influencers highlighted in orange and negative in blue):**

| **Model** | **Cross validation errors** |
| --- | --- |
| M2 – Status_Floating + Bacterial_qPCR | 0.00352 |
| M3 – Status_cDNA + Settling Velocity + Bacterial_qPCR | 0.00364 |
| M1 – Bacterial_qPCR | 0.00365 |

|  | **LCBD – unifrac – M1** | | | | | | | |
| --- | --- | --- | --- | --- | --- | --- | --- | --- |
| *Predictors* | *Estimates* | *std. Error* | *std. Beta* | *CI* | *standardized CI* | *Statistic* | *p* | *df* |
| (Intercept) | 0.01926 | 0.03016 | 0.00000 | -0.04329 – 0.08181 | -0.42751 – 0.42751 | 0.00000 | 0.530 | 22.00000 |
| Bacterial_qPCR | 0.00228 | 0.00307 | 0.15651 | -0.00408 – 0.00865 | -0.28019 – 0.59321 | 0.74326 | 0.465 | 22.00000 |
| Observations | 24 | | | | | | | |
| R^2^ / R^2^ adjusted | 0.024 / -0.020 | | | | | | | |
| ** p<0.05   ** p<0.01   *** p<0.001* | | | | | | | | |

|  | **LCBD – Unifrac – M2** | | | | | | | |
| --- | --- | --- | --- | --- | --- | --- | --- | --- |
| *Predictors* | *Estimates* | *std. Error* | *std. Beta* | *CI* | *standardized CI* | *Statistic* | *p* | *df* |
| (Intercept) | -0.03134 | 0.04018 | 0.00000 | -0.11489 – 0.05221 | -0.40835 – 0.40835 | 0.00000 | 0.444 | 21.00000 |
| Status_Floating | -0.00378 | 0.00210 | -0.51475 | -0.00814 – 0.00058 | -1.10890 – 0.07939 | -1.80172 | 0.086 | 21.00000 |
| Bacterial_qPCR | 0.00763 | 0.00417 | 0.52307 | -0.00104 – 0.01629 | -0.07108 – 1.11722 | 1.83083 | 0.081 | 21.00000 |
| Observations | 24 | | | | | | | |
| R^2^ / R^2^ adjusted | 0.155 / 0.075 | | | | | | | |
| ** p<0.05   ** p<0.01   *** p<0.001* | | | | | | | | |

|  | **LCBD – Unifrac – M3** | | | | | | | |
| --- | --- | --- | --- | --- | --- | --- | --- | --- |
| *Predictors* | *Estimates* | *std. Error* | *std. Beta* | *CI* | *standardized CI* | *Statistic* | *p* | *df* |
| (Intercept) | -0.05158 | 0.05001 | 0.00000 | -0.15590 – 0.05275 | -0.41825 – 0.41825 | 0.00000 | 0.315 | 20.00000 |
| Status_cDNA | -0.00061 | 0.00162 | -0.08257 | -0.00398 – 0.00277 | -0.54296 – 0.37782 | -0.37411 | 0.712 | 20.00000 |
| Settling_Velocity | 0.00020 | 0.00011 | 0.54595 | -0.00003 – 0.00044 | -0.08698 – 1.17888 | 1.79931 | 0.087 | 20.00000 |
| Bacterial_qPCR | 0.00826 | 0.00458 | 0.56688 | -0.00130 – 0.01782 | -0.08887 – 1.22264 | 1.80325 | 0.086 | 20.00000 |
| Observations | 24 | | | | | | | |
| R^2^ / R^2^ adjusted | 0.161 / 0.035 | | | | | | | |
| ** p<0.05   ** p<0.01   *** p<0.001* | | | | | | | | |

**Top models for LCBD using Weighted UniFrac DISTANCES (Model parameters given afterwards with significant positive influencers highlighted in orange and negative in blue):**

| **Model** | **Cross validation errors** |
| --- | --- |
| M1 – Status_cDNA | 0.00750 |
| M2 –Bacterial_qPCR + Archaea_qPCR | 0.00781 |
| M3 – Status_DNA + Linoleic_Acid + Archaea_qPCR | 0.00812 |

|  | **LCBD – Weighted Unifrac – M1** | | | | | | | |
| --- | --- | --- | --- | --- | --- | --- | --- | --- |
| *Predictors* | *Estimates* | *std. Error* | *std. Beta* | *CI* | *standardized CI* | *Statistic* | *p* | *df* |
| (Intercept) | 0.04020 ^***^ | 0.00213 | 0.00000 | 0.03578 – 0.04462 | -0.42378 – 0.42378 | 0.00000 | **<0.001** | 22.00000 |
| Status_cDNA | 0.00294 | 0.00301 | 0.20353 | -0.00331 – 0.00919 | -0.22937 – 0.63642 | 0.97503 | 0.340 | 22.00000 |
| Observations | 24 | | | | | | | |
| R^2^ / R^2^ adjusted | 0.041 / -0.002 | | | | | | | |
| ** p<0.05   ** p<0.01   *** p<0.001* | | | | | | | | |

|  | **LCBD** | | | | | | | |
| --- | --- | --- | --- | --- | --- | --- | --- | --- |
| *Predictors* | *Estimates* | *std. Error* | *std. Beta* | *CI* | *standardized CI* | *Statistic* | *p* | *df* |
| (Intercept) | 0.08956 | 0.05906 | -0.00000 | -0.03327 – 0.21239 | -0.42454 – 0.42454 | -0.00000 | 0.144 | 21.00000 |
| Bacterial_qPCR | -0.01598 | 0.01132 | -0.55688 | -0.03952 – 0.00756 | -1.37736 – 0.26361 | -1.41147 | 0.173 | 21.00000 |
| Archaea_qPCR | 0.01129 | 0.00917 | 0.48553 | -0.00779 – 0.03037 | -0.33495 – 1.30602 | 1.23064 | 0.232 | 21.00000 |
| Observations | 24 | | | | | | | |
| R^2^ / R^2^ adjusted | 0.087 / -0.000 | | | | | | | |
| ** p<0.05   ** p<0.01   *** p<0.001* | | | | | | | | |

|  | **LCBD – Weighted Unifrac – M3** | | | | | | | |
| --- | --- | --- | --- | --- | --- | --- | --- | --- |
| *Predictors* | *Estimates* | *std. Error* | *std. Beta* | *CI* | *standardized CI* | *Statistic* | *p* | *df* |
| (Intercept) | 0.15597 | 0.13211 | 0.00000 | -0.11961 – 0.43155 | -0.43634 – 0.43634 | 0.00000 | 0.252 | 20.00000 |
| Status_DNA | -0.00808 | 0.00680 | -0.55927 | -0.02226 – 0.00610 | -1.54120 – 0.42266 | -1.18809 | 0.249 | 20.00000 |
| Linoleic_Acid | 0.00050 | 0.00112 | 0.18386 | -0.00182 – 0.00283 | -0.66977 – 1.03750 | 0.44929 | 0.658 | 20.00000 |
| Archaea_qPCR | -0.01156 | 0.01363 | -0.49701 | -0.03998 – 0.01687 | -1.71939 – 0.72536 | -0.84814 | 0.406 | 20.00000 |
| Observations | 24 | | | | | | | |
| R^2^ / R^2^ adjusted | 0.087 / -0.050 | | | | | | | |
| ** p<0.05   ** p<0.01   *** p<0.001* | | | | | | | | |

**REFERENCES**

Clarke, K.R., Ainsworth, M., 1993. A method of linking multivariate community structure to environmental variables. Mar. Ecol. Prog. Ser. 92, 205–219.

Darzi, Y., Letunic, I., Bork, P., Yamada, T., 2018. iPath3. 0: interactive pathways explorer v3. Nucleic Acids Res. 46, W510–W513.

Foster, Z.S.L., Sharpton, T.J., Grünwald, N.J., 2017. Metacoder: An R package for visualization and manipulation of community taxonomic diversity data. PLOS Comput. Biol. 13, e1005404.

Jalanka-Tuovinen, J., Salonen, A., Nikkilä, J., Immonen, O., Kekkonen, R., Lahti, L., Palva, A., de Vos, W.M., 2011. Intestinal Microbiota in Healthy Adults: Temporal Analysis Reveals Individual and Common Core and Relation to Intestinal Symptoms. PLoS One 6, e23035.

Kassambara, A., 2018. Machine Learning Essentials: Practical Guide in R. sthda.

Kembel, S.W., Cowan, P.D., Helmus, M.R., Cornwell, W.K., Morlon, H., Ackerly, D.D., Blomberg, S.P., Webb, C.O., 2010. Picante: R tools for integrating phylogenies and ecology. Bioinformatics 26, 1463–1464.

Kuhn, M., 2008. Building predictive models in R using the caret package. J. Stat. Softw. 28, 1–26.

Lahti, L., Shetty, S., Blake, T., Salojarvi, J., 2017. microbiome R package. Tools Microbiome Anal R.

Love, M.I., Huber, W., Anders, S., 2014. Moderated estimation of fold change and dispersion for RNA-seq data with DESeq2. Genome Biol. 15, 550. https://doi.org/10.1186/s13059-014-0550-8

Lüdecke, D., 2018. sjPlot: Data visualization for statistics in social science (R Package Version, 2 [1])[Computer software].

Lumley, T., Miller, A., 2009. Leaps: regression subset selection. R package version 2.9. See http//CRAN. R-project. org/package= leaps.

McMurdie, P.J., Holmes, S., 2013. phyloseq: An R Package for Reproducible Interactive Analysis and Graphics of Microbiome Census Data. PLoS One 8, e61217.

Ning, D., Deng, Y., Tiedje, J.M., Zhou, J., 2019. A general framework for quantitatively assessing ecological stochasticity. Proc. Natl. Acad. Sci. 116, 16892–16898.

Oksanen, J., Blanchet, F., Kindt, R., Legendre, P., Minchin, P.R., O’hara, R., Simpson, G.L., Solymos, P., Stevens, H.H., Wagner, H., 2015. Vegan: community ecology package. R Package version 2.2-1.

Shetty, S.A., Hugenholtz, F., Lahti, L., Smidt, H., de Vos, W.M., 2017. Intestinal microbiome landscaping: insight in community assemblage and implications for microbial modulation strategies. FEMS Microbiol. Rev. 41, 182–199.

Stegen, J.C., Lin, X., Fredrickson, J.K., Chen, X., Kennedy, D.W., Murray, C.J., Rockhold, M.L., Konopka, A., 2013. Quantifying community assembly processes and identifying features that impose them. ISME J. 7, 2069–2079.

Taylor, M., 2017. sinkr: a collection of functions with emphasis in multivariate data analysis.[Internet].

Vass, M., Székely, A.J., Lindström, E.S., Langenheder, S., 2020. Using null models to compare bacterial and microeukaryotic metacommunity assembly under shifting environmental conditions. Sci. Rep. 10, 2455. https://doi.org/10.1038/s41598-020-59182-1

Verster, A.J., Borenstein, E., 2018. Competitive lottery-based assembly of selected clades in the human gut microbiome. Microbiome 6, 186. https://doi.org/10.1186/s40168-018-0571-8
